# Copper removal from synthetic wastewater using the bacterium *Achromobacter xylosoxidans*: biosorption equilibrium, kinetic, thermodynamic and optimizing parameters by Box-Behnken design approach

**DOI:** 10.7717/peerj.21842

**Published:** 2026-10-28

**Authors:** Mustafa A. Fawzy, Mona Alharbi, Hana Sonbol, Sedky H.A. Hassan, Mostafa Koutb, Mohammed Alorabi, Amany G. Ibrahim

**Affiliations:** 1 Biology Department, College of Sciences, Taif University, Taif, Saudi Arabia; 2 Botany and Microbiology Department, Faculty of Science, Assiut University, Assiut, Egypt; 3 Department of Biology, College of Science, Princess Nourah bint Abdulrahman University, Riyadh, Saudi Arabia; 4 Department of Biology, College of Science, Sultan Qaboos University, Muscat, Oman; 5 Department of Biology, Faculty of Science, Umm Al-Qura University, Makkah, Saudi Arabia; 6 Biotechnology Department, College of Sciences, Taif University, Taif, Saudi Arabia; 7 Botany Department, Faculty of Women for Arts, Science and Education, Ain Shams University, Cairo, Egypt

**Keywords:** Copper ion, Response surface methodology, *Achromobacter xylosoxidans* bacteria, Isotherms, Kinetics, Desorption, Regeneration

## Abstract

**Background:**

Heavy metals such as copper are known water pollutants that can be extremely harmful to the environment and pose serious risks to human health and aquatic organisms. The present study aimed to determine the efficiency of *Achromobacter xylosoxidans* isolated from waste soil as a cost-effective biosorbent for the removal of copper (II) (Cu^2+^) ions from synthetic and real wastewater.

**Methods:**

The rate of copper biosorption by the bacterial strain was examined using batch experiments in 100 ml of aqueous solution, in which separate biosorption tests were performed for temperature (20–45 °C), initial pH (4–9), copper concentration (10–240 g L^−1^), contact time (0-150 min), and biosorbent dose (0.25-5.0 g L^−1^). Using the Box-Behnken design and response surface methodology, the biosorption of Cu^2+^ ions by *A. xylosoxidans* biomass was also mathematically modeled to assess the interactive impacts of the independent factors of temperature, bacterial dosage, and initial metal ion concentration.

**Results:**

The analysis of variance (ANOVA) results revealed that the actual data were in line with the predicted data. Under the optimal circumstances of initial copper concentration of 112.35 mg L^−1^, a biosorbent dose of 1.29 g L^−1^, and a temperature of 34.8 °C, the copper biosorption efficiency of *A. xylosoxidans* was estimated to be 80.15%. The chemisorption of copper ions was controlled by second-order kinetic models, while the Langmuir model indicated monolayer biosorption with a maximum biosorption capacity of 106.38 mg g^−1^. Thermodynamic characteristics revealed the spontaneous and endothermic nature of Cu^2+^ biosorption. Desorption and regeneration tests showed that *A. xylosoxidans* could be desorbed with hydrochloric acid (HCl) to recover Cu^2+^ after three successive biosorption-desorption cycles. High efficiency of the bacteria strain (88.55%) was also shown when applied to a real wastewater sample, indicating effective biosorbent performance in complex environments. Based on Fourier-transform infrared spectroscopy (FTIR) and scanning electron microscopy-energy-dispersive X-ray (SEM-EDX) data, it was suggested that the biosorption mechanism of copper ions involved a combination of complexation, electrostatic interactions, and ion-exchange. The efficiency of the strain in adsorbing Cu^2+^ from contaminated synthetic and real wastewater strongly demonstrated the potential of *A. xylosoxidans* as a promising biosorbent in practical applications.

## Introduction

Environmental contamination resulting from a variety of chemicals, including many hazardous heavy metals, is generally considered to be a rapidly growing global concern. These issues pose a severe threat to human health and ecosystems due to their carcinogenic abilities, making them a global environmental concern (Syarifuddin, Suryani & Tahir, 2025). The most common heavy metal that can contaminate the environment through untreated wastewater is copper. Copper can enter the environment through both man-made and natural sources. Reefing melting plants, electroplating, petroleum industry, and other industries are some of the various copper resources (Sonawdekar & Gupte, 2020). The permissible level of copper ions in drinking water has been established by the US Environmental Protection Agency at 1.3 mg/L (USEPA, 1985; USEPA, 1991). According to this statement, ingestion of copper above the recommended amount can result in acute health issues, including kidney and liver damage, anemia, central nervous system issues, and gastrointestinal irritation (Bashir et al., 2021). Nevertheless, reducing copper concentrations to permissible safe levels remains a significant challenge.

Adsorption, ion exchange, electrochemical redox, filtration, and chemical precipitation are the most successful treatment technologies for reducing heavy metals in contaminated wastewater (Fei & Hu, 2023). On the other hand, biosorption is a potential technology for treating heavy metals in polluted water because of its ability to reduce high levels of pollutants to lower concentrations, good selectivity, reusability, cost-effectiveness, easy separation and metal recovery (Hassan et al., 2025; Bashir et al., 2019). Various organisms such as bacteria, yeasts, algae, and fungi, as well as other natural materials, have been widely utilized as biosorbents for heavy metals.

Bacteria are widely distributed in nature, can be produced cheaply on a large scale, have resistance mechanisms, have short generation times and can adapt to various environments. They use various biosorption methods to efficiently eliminate heavy metals from wastewater. Many bacteria, such as *Streptomyces*, *Pseudomonas*, *Micrococcus*, *Bacillus*, and *Escherichia coli*, have been widely recognized for their strong affinity and high efficiency in biosorption of heavy metals (Pham et al., 2022).

*Achromobacter xylosoxidans* used in this study is a Gram-negative, motile, aerobic, non-fermenting rod-shaped bacterium that is commonly found in the environment and primarily causes healthcare-associated infections (Rajni et al., 2023). Studies have revealed that *Achromobacter xylosoxidans* is effective in treating heavy metals such as zinc, nickel, lead, copper, and cadmium. Subudhi et al. (2016) stated that *A. xylosoxidans* could adsorb nearly 90% of copper ions, while Habibollahi et al. (2017) found that *Achromobacter* sp. exhibited a copper biosorption efficiency of 78.31%. In comparison, other bacterial strains showed lower copper removal efficiencies, including *Bacillus amyloliquefaciens* SYNU1 (53.09%), *Pseudomonas stutzeri* (50%), *Microbacterium oxydans* (48.6%), *Geobacillus thermantarcticus* (46.3%), and *Anoxybacillus amylolyticus* (39.8%) (He et al., 2024; Palanivel et al., 2020; Heidari, Mazloomi & Sanaeizade, 2020; Özdemir et al., 2013).

Theoretically, copper (II) (Cu^2+^) ions are predicted to bind to bacterial cells by interacting with surface functional groups acting as potential active binding sites. The surface of *Achromobacter xylosoxidans* can offer an anchor site for the introduction of many functional groups, including hydroxyl groups (−OH), amino groups (−NH), and carboxylic groups (−COOH). The adsorption mechanism between these functional groups and metal ions may be attributed to electrostatic attractions and ion exchange (Gu et al., 2019). Therefore, *A. xylosoxidans* was selected because of its cell wall, which is rich in functional groups, and its effectiveness in ion adsorption, making it a superior choice compared to other bacterial strains.

Despite extensive investigations on bacterial biosorbents, limited research has studied the effectiveness of an indigenous *Achromobacter xylosoxidans* strain isolated from waste soil in Jeddah’s Second Industrial City, KSA, for copper removal as a cost-effective and eco-friendly biosorbent. By integrating multi-parameter optimization using a response surface methodology with adsorption modeling and possible mechanistic analysis, which has not been widely discussed for this specific strain, this study presents a novel and scientifically relevant advance in the field of wastewater treatment. From an engineering perspective, although synthetic wastewater was initially employed to manage experimental conditions, the bacterial performance was further confirmed using real wastewater. The stability and practical application of the biosorbent were evidenced by bacterial regeneration and recycling tests, offering benefits such as cost-effectiveness, operational stability, and simplicity in handling for possible large-scale applications.

As a result, the study aimed to examine the biosorption efficiency of copper ions by *A. xylosoxidans*. Response surface methodology and Box-Behnken Design were employed to optimize factors affecting copper biosorption. To study the biosorption thermodynamics, kinetics, and isotherms, the impacts of temperature, pH, bacterial dose, contact time, and Cu^2+^ concentration were also assessed through batch experiments. Biosorption/desorption tests evaluated biomass regeneration feasibility, and Cu^2+^ removal from real wastewater were also performed. Finally, the potential biosorption mechanisms were examined using Fourier transform infrared spectroscopy (FTIR), and scanning electron microscopy-energy-dispersive X-ray (SEM-EDX) techniques, providing a deeper understanding that goes beyond simply optimizing the performance of the biosorption process. The findings are predicted to contribute to the development of efficient and sustainable technologies for wastewater treatment.

## Materials & Methods

### Cultivation and preparation of dried bacterial biomass

*Achromobacter xylosoxidans* strain was isolated from waste soil in the Second Industrial City of Jeddah, KSA. The isolated bacterium was cultured in 250 ml conical flasks that contained 0.5% peptone, 0.3% yeast extract, and 0.5% NaCl at pH 7, and incubated in an incubator at 37 °C with shaking for 24 h (Ilesanmi et al., 2020). Following the incubation, the bacterial samples were centrifuged at 10,000 rpm for 10 min, washed twice with 0.9% NaCl, and then dried in an oven at 80 °C for 24 h. The dried biomass was then ground and subjected to autoclaving at 121 °C for 15 min to examine cell death. The powdered cells were then inoculated into fresh liquid medium, and the lack of any growth showed that the bacteria were no longer alive.

### Characterization of biomass

Scanning electron microscopy energy equipped with a dispersive X-ray device (SEM-EDX, VEGAII, XMU, TESCAN, Faculty of Science, Alexandria University) were employed to examine the surface morphology of *A. xylosoxidans*. The dried samples were pelleted with KBr and the spectra of Fourier transform infrared were obtained using a Shimadzu FT-IR-8400S spectrometer, Kyoto, Japan. The spectrum of the dried samples was obtained over a wave number range of 500 to 4,000 cm^−1^.

### Preparation of copper solution

The aqueous stock solution of copper sulfate (CuSO_4_.5H_2_O) was freshly prepared by dissolving about 3.93 g in 1 L of distilled water. To ensure complete dissolution of the copper, the stock solution was agitated for fifteen minutes and then left to stand for twenty-four hours, after which the solution was kept at 25 °C.

### Biosorption studies

Biosorption experiments were carried out in 100 mL of aqueous solution. The pH was adjusted to values ranging from 4 to 9 using 0.1 M NaOH and hydrochloric acid (HCl) to estimate the impact of pH on the biosorption capacity and uptake efficiency of copper ions by *A. xylosoxidans* bacteria. Temperatures of 20, 25, 30, 35, 40, and 45 °C and biosorbent dosages of 0.25, 0.5, 1.25, 2.5, and 5.0 g L^−1^ were used to assess their effect on copper ion removal. Additionally, to identify the equilibrium time, the impact of contact time from 0 to 150 min was also studied. The impact of initial copper concentration on the biosorption process was examined by varying the copper concentration to 10, 30, 60, 90, 120, and 240 mg/L. In each experiment, the investigated factor was varied, while other parameters remained constant (bacterial dose: 1 g L^−1^, pH: 6, initial Cu^2+^ concentration: 50 mg L^−1^, contact time: 60 min, and temperature: 30 °C), and the solution was stirred at 120 rpm. Each trial was conducted twice, and the biosorbent was separated from the liquid phase by centrifugation. The residual concentration of Cu^2+^ ion in the solution was measured using an Inductively Coupled Plasma-Optical Emission Spectrometer (ICP-OES; Perkin Elmer Optima 2000DV).

The copper uptake efficiency (R, %) and biosorption capacity (q, mg/g) were determined using the following formulas:

(1) \begin{eqnarray*}\mathrm{R}& = \frac{{C}_{0}-{C}_{eq}}{{C}_{0}} \times 100\end{eqnarray*}

(2) \begin{eqnarray*}q& = \frac{ \left( {C}_{0}-{C}_{eq} \right) V}{m} \end{eqnarray*}

where C_0_ and C_eq_ represent the initial and equilibrium copper concentration (mg L^−1^), respectively; m is the biosorbent’s mass (g); and V (mL) is the volume of aqueous solution.

### Experimental design

To assess the impact of independent parameters such as copper ion concentration (mg L^−1^), bacterial dose (g L^−1^) and temperature (^∘^C) on the efficiency of copper ion removal by bacterial isolate and to estimate the optimal response value, response surface methodology (RSM) through Box–Behnken design (BBD) was used.

Each parameter had three levels: low, medium and high, with coded values of −1, 0, and +1, respectively. The BBD matrix is displayed in Table S1, along with the ranges and levels (actual and coded) of the three variables. Fifteen trials were conducted at three levels and three replicates at the center point according to the interaction of these parameters. The experimental results were processed using Design-Expert software version 7 (Stat-Ease Inc., Minneapolis, USA). Regression analysis was performed to determine the response function coefficients of the experimental results. The adequacy of suggested quadratic model and the significance of the independent parameters effects were assessed by analysis of variance (ANOVA).

### Thermodynamic studies

To evaluate the thermodynamic parameters, biosorption tests were performed at four temperatures (293, 298, 303, and 308 K) with fixed parameters of 1 g L^−1^ bacterial dose, pH 6, 50 mg L^−1^ initial Cu^2+^ concentration, and 60 min exposure time. Finally, the Cu^2+^ ion concentration in the solution was measured as described above.

### Isotherm model studies

To investigate the isothermal copper biosorption, approximately 100 mL of copper ion solutions with levels ranging from 10 to 240 mg L^−1^ were used at fixed parameters (bacterial dose = 1 g L^−1^, pH = 6, contact time = 60 min, and temperature = 30 °C). The biosorption isotherm of Cu^2+^ ions by the bacterial strain was analyzed using the Freundlich, Langmuir, and Dubinin–Radushkevich isotherm models.

### Kinetic model studies

Kinetics experiments were conducted at various time intervals ranging between 0 and 150 min under constant conditions of bacterial dose (1 g L^−1^), pH (6), temperature (30 °C), and initial copper concentration (50 mg L^−1^). The biosorption kinetics were studied using pseudo-first-order, pseudo-second-order, Elovich, and intra-particle diffusion models.

### Desorption and reuse of biosorbent

Four adsorption/desorption cycles were performed using 1 g L^−1^ of bacterial strain with 100 ml of 50 mg L^−1^ Cu^2+^ solution at 30 °C, pH 6 and 120 rpm for 1 h to evaluate biomass reusability. The biomass was then separated by centrifugation, washed with distilled water, and dried at room temperature. Desorption of copper ions was then conducted using 100 mL of 0.1 M HCl for 1 h, followed by thorough washing before reuse. The biosorption-desorption cycle was repeated four times to evaluate the durability and reusability of the bacterial strain, and copper concentration was determined after each cycle.

### Applications on real wastewater sample

A sample of real wastewater was obtained from domestic wastewater in Taif, Saudi Arabia. The experiment was conducted in 100 mL of water sample at a pH 6 for 60 min under optimal conditions, which included copper concentration of 112.35 mg L^−1^, a biosorbent dosage of 1.29 g L^−1^, and a temperature of 34.8 °C. The mixture was agitated at 120 rpm, then the biosorbent was removed by centrifugation, and the copper solution sample was analyzed. Other characteristics of the water sample, such as temperature, pH, total dissolved solids, and electrical conductivity were also assessed.

## Results and discussion

### Biosorption performance of *Achromobacter xylosoxidans*

#### Effect of temperature

The chemical composition of the solutions is influenced by temperature; so, differences in temperature can significantly affect the biosorption rate (Smoczyński, Pierożyński & Mikołajczyk, 2020). Figure 1A displays the results of copper biosorption by *A. xylosoxidans* at different temperatures between 20 °C and 45 °C. The results indicate that the biosorption of copper ions by *A. xylosoxidans* increased with increasing temperature up to a moderate temperature of 30 °C, at which maximum biosorption capacity and uptake efficiency (39.82 mg/g and 79.64%, respectively) were achieved. This is attributed to increased molecular motion and interaction between copper ions and the binding sites on the bacterial biomass. In this context, Xie et al. (2021) found that the copper biosorption rate of *Deinococcus wulumuqiensis* increased with temperature, reaching a peak of 77.6% at 32 °C. However, at temperatures above 30 °C, a decrease in the rate of biosorption was observed, which can be attributed to the effects of desorption or the disruption of binding interactions resulting from thermal agitation (Alharbi et al., 2025). In addition, increased temperature reduces the biosorption rate of ions due to physical damage to the adsorbent (Farmanbordar et al., 2021). Therefore, the biosorption process can usually be carried out at room temperature because it is highly reproducible.

**Figure 1:**
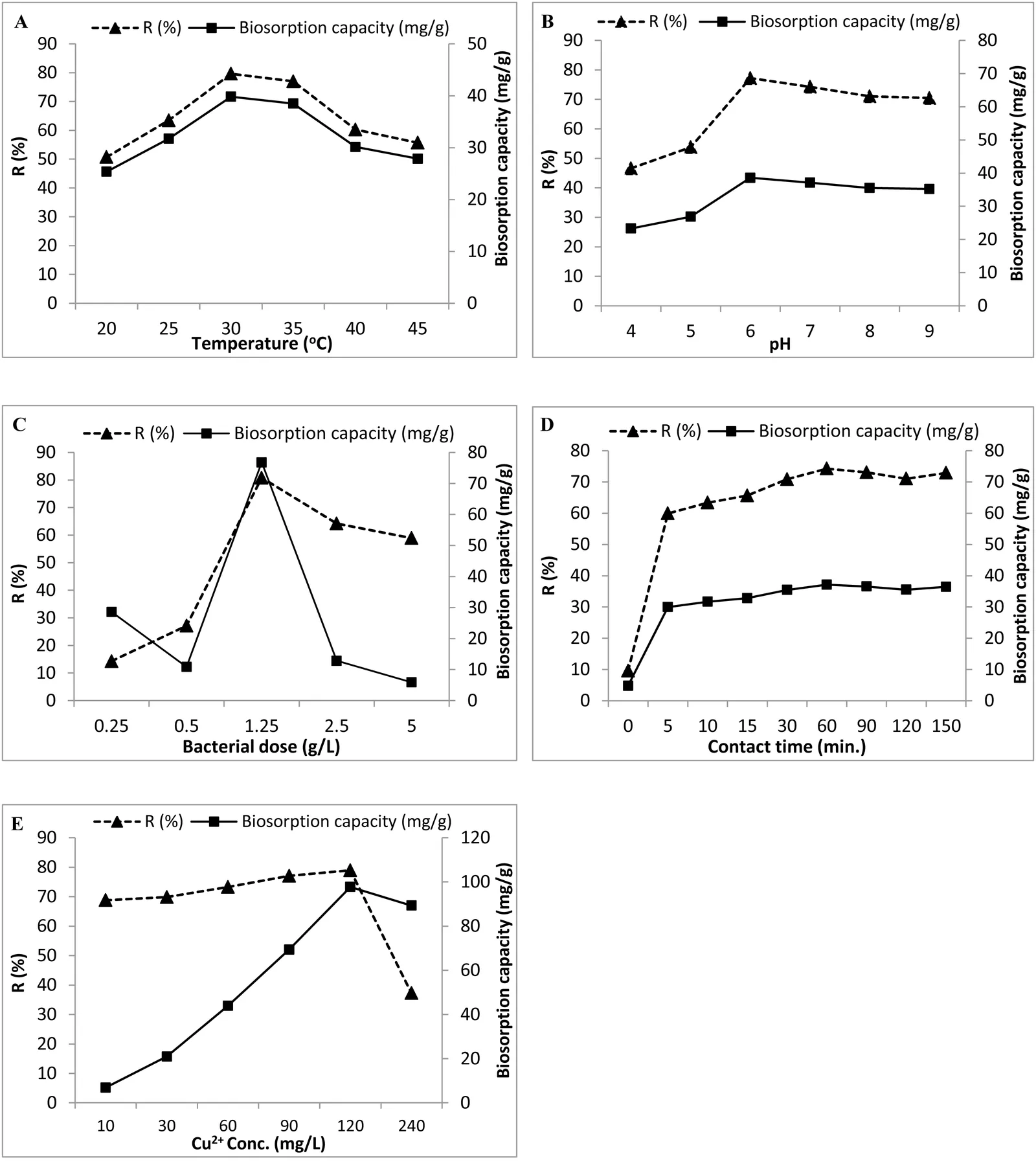
Bioremoval efficiency (%) and biosorption capacity (mg/g) of copper ions by *A. xylosoxidans*. (A) At various temperatures, (B) at different pH values, (C) at various bacterial doses, (D) at various contact time, and (E) at different initial copper concentrations.

#### Effect of pH

The pH level of a solution is a key limiting factor in the biosorption of heavy metals, influencing the availability of the binding sites of metals on the surface of biosorbent and the chemical properties of the metals in solution (Aloufi et al., 2024). Figure 1B indicates the impact of pH on the biosorption of Cu^2+^ ions by *A. xylosoxidans* biomass. It is worth noting that at pH 4, the biosorption efficiency and capacity are low, but they gradually increase to 77.2% and 38.6 mg/g, respectively, at pH 6.

The low efficiency of metal ion biosorption at low pH is due to competitive between metals and hydrogen ions. Shuhong et al. (2014) also found that as the pH decreases, hydrogen ions (H^+^) block the biosorption sites of the cell wall, thereby generating repulsive forces that prevent metal ions from reaching the surface functional groups. At elevated pH (6), deprotonation of these functional groups imparts a net negative charge to the bacterial surface. This phenomenon may enhance the electrostatic interaction between copper ions and the bacterial surface (Al-Zaban et al., 2022). This suggests that the optimal pH for Cu^2+^ biosorption by *A. xylosoxidans* is 6. Similar studies have found that the optimal pH for copper ion removal by *Rhodococcus erythropolis* is 6.0 (Baltazar et al., 2019). When the pH increases to the alkaline range (>6), it tends to form and precipitate metal hydroxides, which results in a decrease in the biosorption rate at higher pH values (Fawzy et al., 2023). As a result, biosorbents typically achieve maximum ion removal at slightly acidic pH values, usually in the range of 5.0 to 6.25 (Xie et al., 2021).

#### Effect of biosorbent dosage

The ratio of adsorbent dosage to metal ions is the important parameter in the biosorption process of adsorbents. Figure 1C illustrates that the Cu^2+^ removal percentage and biosorption capacity of *A. xylosoxidans* increased significantly with increasing bacterial dosage, achieving a peak of 80.78% with a biosorption uptake of 76.78 mg g^−1^ at a biosorbent dose of 1.25 g L^−1^. This is due to more exchangeable active sites available for copper ions. Thus, *A. xylosoxidans* achieved the highest Cu^2+^ biosorption at a biosorbent dose of 1.25 g L^−1^. However, at a given metal concentration, no further increase in biosorbent amount was required to ensure adequate biosorption. The rapid biosorption of Cu^2+^ ions onto the surface of the bacterial biomass leads to a reduction in the available binding sites, decreasing removal efficiency and biosorption capacity of *A. xylosoxidans* (Ayub et al., 2021).

Higher biosorbent dosage leads to a sieving impact of the dense outer layer, which hinders the access of copper ions to available biosorption sites, and thus reduces the amount of copper biosorpiton per unit of bacterial biomass (Fakhry et al., 2013).

#### Effect of contact time

The contact time between metal ions and adsorbents is a key variable influencing the biosorption process. The results depicted in Fig. 1D show that the biosorption efficiency and capacity of Cu^2+^ ions by *A. xylosoxidans* increased rapidly during the first 10 min, reaching above 63% and 31 mg/g, respectively. After this initial period, the biosorption efficiency and uptake capacity increased slightly and continued to rise until reaching equilibrium after 60 min, reaching 74.28% and 37.14 mg/g, respectively. Following this point, the uptake of copper by *A. xylosoxidans* remained nearly constant (Fig. 1D). Therefore, the biomass demonstrated gradual biosorption during the early stages of contact with copper (Cu) ions. Biosorption initially occurred on the outer surface of the adsorbent until saturation was reached. As the contact time increased, the metal began to diffuse into the internal pores of the material, occupying the available inner biosorption sites, which could slow down the rate of biosorption (Liu et al., 2021). Our findings are in agreement with those stated by Farmanbordar et al. (2021), who found that copper biosorption by *Bacillus thuringiensis*, *Bacillus subtilis*, and *Bacillus pumilus* reached equilibrium after 60 min.

#### Effect of initial copper concentration

This study demonstrated that the Cu^2+^ removal percentage and biosorption capacity of *A. xylosoxidans* increased from 68.8% to 78.9% and from 6.88 mg g^−1^ to 94.72 mg g^−1^, respectively, as the initial Cu^2+^ concentration increased from 10 mg L^−1^ to 120 mg L^−1^ (Fig. 1E). This increase can be explained by the increased driving force brought about by the increased ion concentration and the increased availability of binding sites on the outer surface of the bacterial biomass under conditions of reduced competition (Chintalapudi et al., 2022).

When the bacterial biomass reaches the saturation state of Cu ions, further increasing the metal concentration does not increase the amount of adsorbed copper. In other words, the maximum uptake of copper was observed at an initial concentration of 120 mg L^−1^ and then decreased (reached a steady state) with increasing copper concentration. This is due to the saturation of available binding sites and competition of large amounts of copper ions for free biosorption sites on the surface of bacterial biomass. These data are in agreement with the results of Sonawdekar & Gupte (2020) who reported that *Bacillus cereus* sys1 removed about 73% of copper at a concentration of 100 mg L^−1^ and that the uptake decreased with increasing Cu^2+^ concentration. Alam & Ahmad (2013) also found that the highest biosorption capacity of *Exiguobacterium* sp. ZM-2 for copper ions reached about 19.9 mg/g at a concentration of 100 mg L^−1^.

### Optimization of experimental parameters using a Box–Behnken design

The response surface methodology incorporating Box–Behnken design consisted of 15 experiments with three parameters and three levels, during which the initial Cu^2+^ concentration (10–120 mg L^−1^), bacterial dose (1.25–5 g L^−1^), and temperature (20–40 °C) were varied while maintaining a contact time of 60 min and a pH of 6. As shown in Table S1, the removal rates of copper from aqueous solutions by *A. xylosoxidans* ranged from 64.40% to 82.40%. Among the 15 BBD runs, the results of run 2 were the most outstanding, with the highest removal percentage of 82.40%.

#### ANOVA analysis

The obtained data were evaluated using analysis of variance (ANOVA), and based on this, the best-fit model for predicting the biosorption efficiency of copper ions by *A. xylosoxidans* bacteria was proposed, as presented in Table S2. The data showed that the model was significant with a *p* value of 0.009 and *F* value of 10.22, but the lack-of-fit was not significant (*p* = 0.847). This indicates that the quadratic model is suitable for predicting the copper removal efficiency of *A. xylosoxidans*. Additionally, the coefficient of determination (R^2^) for copper removal percentage was 0.948, showing that the model is able to adequately represent the relationship between the selected variables. The low coefficient of variation (CV) value (2.78%) shows minimal variation in the mean value, indicating that the system studied has a high repeatability (Zar, 2010).

The ANOVA data in Table 1 show the impacts of independent process factors on the Cu^2+^ removal efficiency as linear, interactions, and quadratic terms. As can be seen from Table 1, the only terms that significantly affected the Cu^2+^ removal percentages by *A. xylosoxidans* are the linear term (A) of the initial Cu^2+^ concentration, the interaction term (BC) of bacterial dosage and temperature, and the quadratic term (B^2^) of bacterial dose (*p* values are 0.017, 0.035, and 0.001, respectively). However, the other linear terms (B, and C), interaction terms (AB, and AC), and quadratic terms (A^2^, and C^2^) are not significant (*p* > 0.05).

**Table 1:** Analysis of variance (ANOVA) for the model coefficients of copper ion bioremoval by *A. xylosoxidans*.

	Coefficient estimate	Degree of freedom	SE	*F*	*p*
Intercept	67.526	1	1.16	–	–
**Linear terms**					
A-Cu(II) conc.	2.51	1	0.71	12.40	0.017
B-Bacterial dose	−1.66	1	0.71	5.46	0.067
C-Temp.	0.59	1	0.71	0.69	0.45
**Interaction terms**					
AB	0.89	1	1.01	0.77	0.420
AC	2.54	1	1.01	6.37	0.053
BC	−2.85	1	1.01	8.03	0.035
**Quadratic terms**					
A^2^	2.67	1	1.05	6.47	0.052
B^2^	7.52	1	1.05	51.41	0.001
C^2^	−1.03	1	1.05	0.97	0.369

After eliminating insignificant terms, the following quadratic polynomial Eq. (3) can be used to characterize the impacts of the independent process variables such as initial Cu^2+^ concentration (A), bacterial dosage (B), and temperature (C), on the copper biosorption efficiency (Y).

(3) \begin{eqnarray*}\mathrm{Y}=67.53+2.51\mathrm{A}-1.66\mathrm{B}+0.59\mathrm{C}+0.89\mathrm{AB}+2.54\mathrm{AC}-2.85\mathrm{BC}+2.67{\mathrm{A}}^{2}+7.52{B}^{2}\nonumber\\\displaystyle -1.03{C}^{2}.\end{eqnarray*}

#### Response surface plots of Cu^2+^ biosorption efficiency by *A. xylosoxidans*

As mentioned earlier, the analysis of variance revealed that among the individual parameters, only the linear term of the initial Cu^2+^ concentration had a significant impact on the biosorption efficiency of Cu^2+^ ions; therefore, a one-factor graph was constructed for this factor (Fig. 2). Furthermore, since the mutual effect of bacterial dosage and temperature (BC) was found to be the only significant factor among the interaction variables, a three-dimensional response surface plot and a corresponding contour graph were plotted (Fig. 2).

According to the data in Fig. 2, the biosorption efficiency of copper by *A. xylosoxidans* was positively affected when the Cu ion concentration increased from 10 to 120 g L^−1^. This is because higher metal ion concentrations increase the probability of heavy metal ions colliding with the binding sites of the biosorbent, leading to more efficient biosorption (Karnwal, 2024).

**Figure 2:**
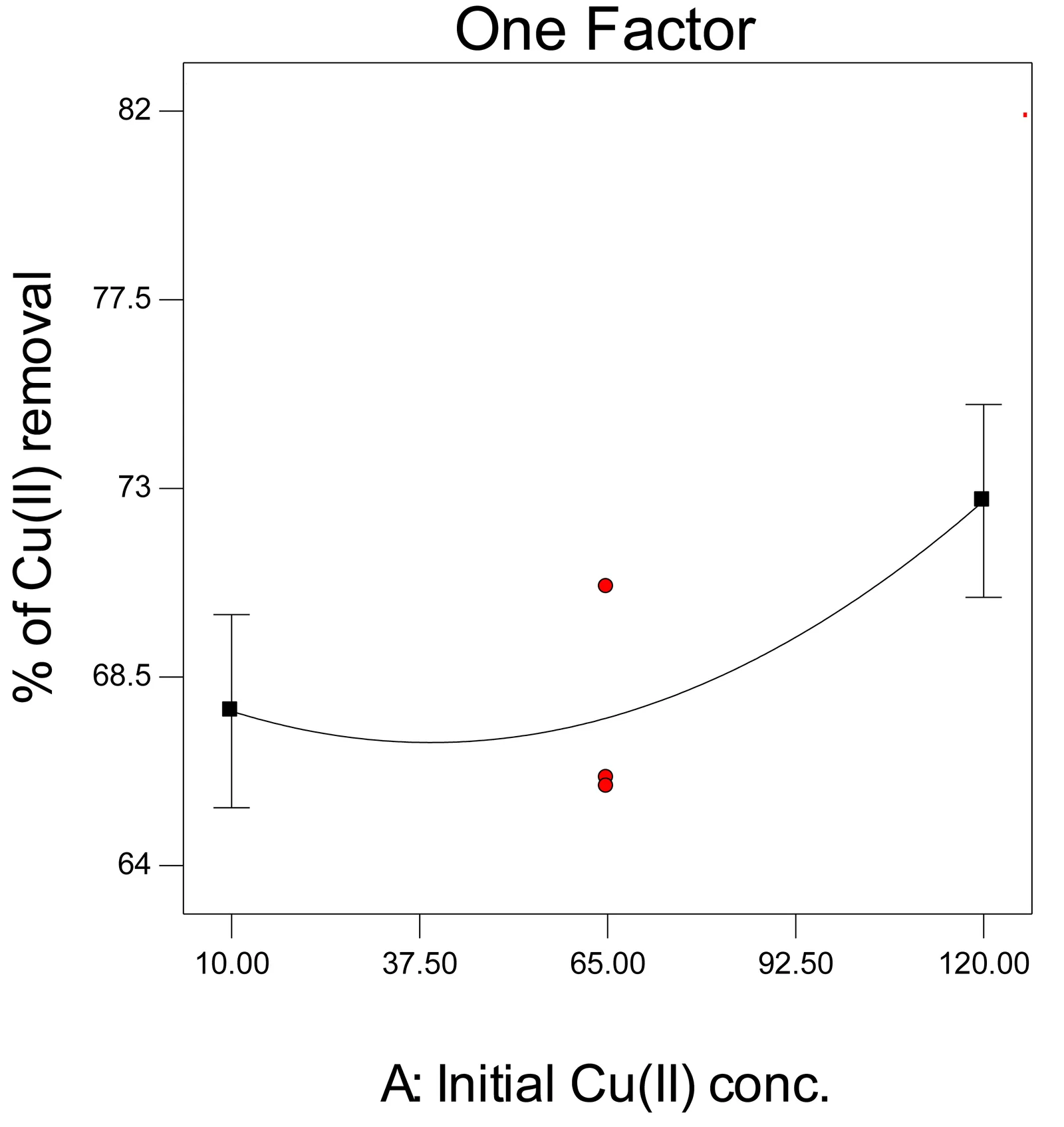
One factor plot of initial Cu(II) conc. on bioremoval efficiency by *Achromobacter xylosoxidans*.

Figure 3 shows 3D response surface and contour plots of the copper removal efficiency as a function of bacterial dosage and temperature at a fixed initial copper concentration (65 mg L^−1^). According to the Figure, the highest percentage of copper removal was recorded at the highest and lowest bacterial dosages. Nevertheless, the biosorption efficiency increased with the gradual increase in temperature, reaching a peak at around 35 °C (74.69%). This is because copper ions have higher biosorption energy at higher temperatures, which facilitates their interaction with the biosorption sites, thereby increasing their biosorption effectiveness. These findings are consistent with previous research results (El-Ahwany, 2012).

**Figure 3:**
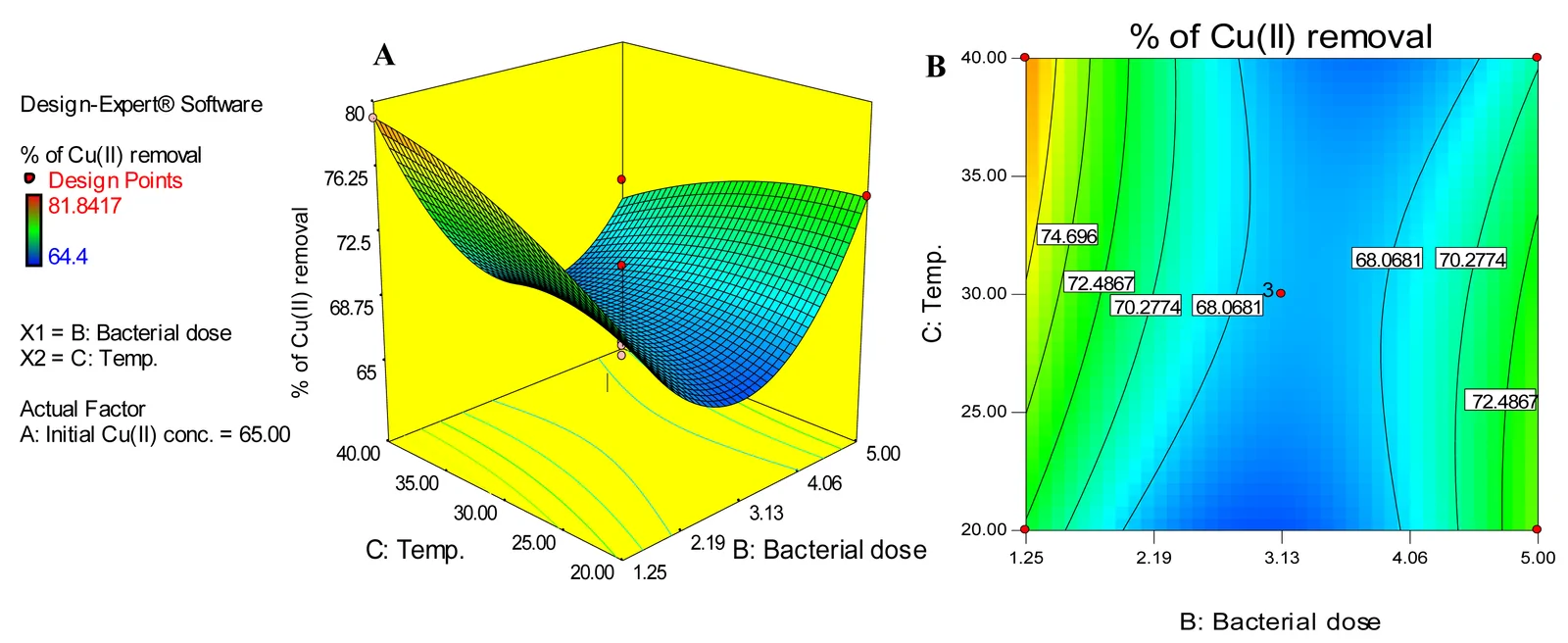
(A) RSM 3D and (B) contour plots displaying the impact of bacterial dosage and temperature on bioremoval efficiency of copper ions (R, %) on *A. xylosoxidans*.

#### Optimization using the desirability function

The desirability function approach was used in order to determine the optimal circumstances for laboratory trials to confirm the applicability and efficiency of the suggested model. Table S3 shows that the experimental results closely match the predicted values for copper removal efficiency, demonstrating the validity of the proposed model. The data indicate that the biosorption efficiency is highest when the initial Cu^2+^ concentration is 112.35 mg L^−1^, the biomass concentration is 1.29 g L^−1^, the temperature is 34.8 °C, and the desirability value is 1.00. The assessed function can accurately express the polynomial quadratic model and the desired circumstances, according to the desirability of 1.00. Under these circumstances, the biosorption efficiency of copper by *A. xylosoxidans* was 80.15%. These findings demonstrate the possibility of maximizing the biosorption efficiency of copper by *A. xylosoxidans* using response surface methodology prediction techniques.

### Biosorption thermodynamics

Thermodynamic analysis of the biosorption process was conducted to determine the energy-dependent mechanism of Cu^2+^ ion biosorption by *A. xylosoxidans*. The equations, parameters and plot of the thermodynamic analysis are listed in Table 2, Fig. S2. It is important to note that the equilibrium constant (Kc) was determined using biosorption data (q_e_/C_e_) and transformed into a dimensionless form by unit correction, taking into account the standard state concentration (water density, 1000 g/L) (Milonjić, 2007). This guarantees the accuracy of the calculated parameters and that Kc is a dimensionless constant.

**Table 2:** Thermodynamic parameters of copper ions bioremoval by *A. xylosoxidans*.

Mathematical Equation	Thermodynamic parameters	Temperature (K)			
		293K	298K	303K	308K
${\mathrm{K}}_{\mathrm{C}}= \frac{q}{{C}_{eq}} \times \rho $	ΔG^o^ (kJmol^−1^)	−16.89	−18.45	−20.80	−20.75
ΔG^∘^ = − RTlnK_C_	Δ*S*^o^ (kJmol^−1^ K^−1^)	0.282			
${\mathrm{lnK}}_{\mathrm{C}}= \frac{\Delta {\mathrm{S}}^{\circ }}{\mathrm{R}} - \frac{\Delta {\mathrm{H}}^{\circ }}{\mathrm{RT}} $	ΔH^o^ (kJmol^−1^)	65.48			
	*r* ^2^	0.84			

**Notes.**

Kc: dimensionless equilibrium constant; *ρ*: the solution density (1,000 g/L); ΔG^o^: Gibb’s free energy; ΔS^o^: entropy; ΔH^o^: enthalpy; R: ideal gas constant (8.31 J/mol K); and T: Temperature in Kelvin.

The high positive enthalpy value (ΔH^∘^ = 65.48 kJ/mol) indicates the endothermic manner of the biosorption and the possibility of physical or chemical biosorption. Deng et al. (2006) found that a chemical biosorption is indicated by a magnitude of 20.9 < ΔH^∘^ < 418.4, confirming that the biosorption of copper ions onto *A. xylosoxidans* biomass was chemisorption. According to Ofudje et al. (2024), such high enthalpy value indicates that strong interactions between the adsorbent and the adsorbate occur during the copper biosorption process, requiring more energy.

The negative Gibb’s free energy (ΔG^o^) values validate the thermodynamic feasibility and spontaneity of copper binding to *A. xylosoxidans* bacteria. Further evidence that biosorption is more feasible at higher temperatures and that there is a greater driving force was provided by the decrease in ΔG^∘^ values as the temperature increased (Ofudje et al., 2020).

The spontaneous biosorption of Cu^2+^ ions onto the surface of *A. xylosoxidans* is further supported by entropy (ΔS^∘^ = 0.282 kJ/mol K), reflecting an increase in randomness during the biosorption process (Hady et al., 2025). Moreover, the positive ΔS^∘^ change indicates a strong affinity between the available biosorption sites on the *A. xylosoxidans* surface and copper ions.

### Biosorption isotherms

Biosorption isotherm models are widely used to explain the biosorption mechanism, biosorption affinity, and surface characteristics of adsorbents. To investigate the retention mechanism of Cu^2+^ ions on *A. xylosoxidans* biomass, three isotherm models such as the Langmuir, Freundlich, and Dubinin–Radushkevich were studied. Table 3 shows the equations and parameters derived from these models.

**Table 3:** Isotherm and kinetic parameters of copper ions bioremoval by *A. xylosoxidans*.

**Models**	**Equations**	**Isotherm parameters**		**Values**
Langmuir	$ \frac{{C}_{eq}}{q} = \frac{1}{{q}_{max}b} + \frac{{C}_{eq}}{{q}_{max}} {\mathrm{R}}_{L}=1/(1+{K}_{L}{C}_{o})$	*r* ^2^		0.926
		*q*_*max*_ (mg/g)		106.38
		*b* (L/mg)		0.04
		*R*_*L*_		0.086–0.417
Freundlich	$ln~q=ln~{K}_{F}+ \frac{1}{n} ~Ln~{C}_{eq}$	*r* ^2^		0.731
		*K*_*f*_ (mg/g)		5.46
		*n*		1.47
Dubinin-Radushkevich	*ln* *q* = *lnq*_0_ − *βɛ*^2^	*r* ^2^		0.804
	$=RT\mathit{ln}(1+ \frac{1}{{C}_{eq}} )$	*q*_*o*_ (mg/g)		63.75
		*β* × 10^−6^ (mol^2^/J^2^)		5.0
	$E=\sqrt{1/2}\beta $	*E* (kJ/mol)		0.316
**Models**	**Equations**	**Kinetic parameters**		**Values**
Experimental data		*q* (exp.) (mg/g)		37.14
PFO	$log \left( q-{q}_{t} \right) =logq- \frac{{K}_{1}t}{2.303} $	*r*^2^*q* (cal.) (mg/g) *K*_1_ (min^−^^1^)		0.835 17.22 0.085
PSO	$ \frac{t}{{q}_{t}} = \frac{1}{{K}_{2}{q}^{2}} + \frac{t}{q} $	*r*^2^*q* (cal.) (mg/g) *K*_2_(g/mg min)		0.999 36.63 0.027
Elovich	${q}_{t}= \frac{1}{\beta } \ln \left( \alpha \beta \right) + \frac{1}{\beta } \ln \left( \mathrm{t} \right) ~$	*r*^2^*α* (g/mg min) *β* (g/mg)		0.811 2.108 0.135
		Zone 1	*r* ^2^	0.798
		Zone 2	*r* ^2^	0.752
		Zone 1	*K*_*i*_(mg/g min^1/2^)	5.386
Intra-particle diffusion	*q*_*t*_ = *K*_*i*_*t*^0.5^ + *C*_*i*_	Zone 2	*K*_*i*_(mg/g min^1/2^)	0.131
		Zone 1	*C*_*i*_(mg/g)	8.675
		Zone 2	*C*_*i*_(mg/g)	32.51

**Notes.**

For isotherm parameters, q and q_max_: the equilibrium and maximum biosorption capacity, respectively; b: Langmuir isotherm constant; C_0_: the initial copper concentration; R_L_: the separation factor; n: the adsorption intensity; K_F_: the adsorption capacity; *ɛ*: polyani potential; q_o_: the theoretical saturation capacity of the adsorbent; E: the mean free energy of biosorption; and β: the adsorption energy. For kinetic parameters, q_t_ is the biosorption capacity at time; k_1_ and k_2_ are the rate constants of pseudo-first-order and pseudo-second-order and intra-particle diffusion models, respectively; α is the initial rate of adsorption; *β* is the desorption constant; k_i_ is the rate constant of intra-particle diffusion model; C_i_ is the constant of boundary layer thickness; 1 and 2 are the zone 1 and 2 of the linear plots of intra-particle diffusion model.

The high correlation coefficient (*r*^2^ > 0. 926) indicated that the Langmuir isotherm model was a good fit for the biosorption of copper by *A. xylosoxidans* (Fig. S3A; Table 3). Furthermore, the biomass of *A. xylosoxidans* exhibited the greatest copper binding capacity (q_max_ = 106.38 mg/g) with a Langmuir constant (*b*) of 0.04 L/mg.

A comparative analysis of the biosorption of Cu^2+^ ion using *A. xylosoxidans* with other previously published investigations based on the maximum biosorption capacity (q_max_) is shown in Table 4. Therefore, based on this comparison, *A. xylosoxidans* is an effective biosorbent for the removal of Cu^2+^ ions from wastewater.

**Table 4:** Comparison of copper biosorption capacities of *A. xylosoxidans* with other biosorbents.

Biosorbents	q_max_ (mg/g)	Reference
*Pseudomonas aeruginosa*	70.4	Zolgharnein et al. (2010)
*Bacillus thioparans*	27.3	Rodríguez-Tirado, Green-Ruiz & Gómez-Gil (2012)
*Bacillus subtilis*	100	Liu et al. (2021)
*Exiguobacterium* sp. ZM-2	20.1	Alam & Ahmad (2013)
Lyophilized *Pseudomonas aeruginosa*	60.3	Kőnig-Peter, Kilar & Pernyeszi (2019)
Lyophilized *Pseudomonas fluorescens*	56.0	Kőnig-Peter, Kilar & Pernyeszi (2019)
*Rhodococcus erythropolis*	68.06	Baltazar et al. (2019)
*Moringa oleifera*	23.3	Tokay & Akpınar (2021)
*Deinococcus wulumuqiensis*	1.25	Xie et al. (2021)
*Bacillus subtilis* OQ931871	19.88	Danial & Dardir (2023)
*Bacillus pumilus* OQ931870	11.876	Danial & Dardir (2023)
*Pseudomonas alcaliphila* GKSM11	31.15	Majhi et al. (2023)
*Bacillus stercoris* GKSM6	35.05	Majhi et al. (2023)
*Achromobacter xylosoxidans*	106.38	This study

The Langmuir constant (*b*) reflects the affinity of the biosorbent for Cu^2+^ ions and is used to determine the dimensionless separation factor (*R*_*L*_). When R_L_ is between 0 and 1, biosorption is favorable, while when *R*_*L*_ >1, it is unfavorable (Nnaji et al., 2021). In the present study, the *R*_*L*_ values ranged from 0.086 to 0.417, suggesting a good biosorption process (Table 3).

Compared with the Langmuir model, the Freundlich isotherm model showed a lower correlation coefficient (*r*^2^ = 0.731; Fig. S3B), suggesting that the isotherm data did not correlate well with the model. Moreover, higher value of biosorption intensity (*n* = 1.47; Table 3) (greater than 1) indicates more favorable biosorption conditions and the formation of strong interactions between Cu^2+^ ions and *A. xylosoxidans* biomass (Soleymani et al., 2023). Similar conclusions were drawn by Sonawdekar & Gupte (2020) on the biosorption of Cu^2+^ ions by *Bacillus* cereus sys1. According to their research, *Bacillus cereus* cells indicated adequate biosorption when the biosorption intensity (*n*) was greater than 1, which was also the case in the current investigation.

Considering the Dubinin–Radushkevich model (Fig. S3C), the chemisorption or physisorption of the biosorption process can be explained by the *E* value (average free energy of biosorption). As stated by Atkins & de Paula (2006), *E* values below 8 kJ/mol show that the biosorption process is physical in nature. In this study, the low *E* value (0.316 kJ/mol; Table 3), showing that the biosorption of copper by *A. xylosoxidans* is controlled by a physical mechanism, such as weak van der Waals forces and electrostatic attractions.

From the above results, it can be concluded that the Langmuir isotherm model can more clearly describe the biosorption copper by *A. xylosoxidans* than other models. This shows that the surface of bacteria is homogeneous and the cations are biosorbed on the surface of biosorbent in the form of a monolayer (Mahdi et al., 2023). Furthermore, this monolayer biosorption is important because it increases the interaction between copper ions and the bacterial surface, thereby improving the effectiveness of the biosorption process. These findings support the use of the Langmuir model to describe the biosorption properties of *Achromobacter* bacteria.

### Biosorption kinetics

Biosorption kinetics controls the biosorption rate, which is a crucial component in the design of biosorption systems because it offers relevant details about the biosorption mechanisms and reaction pathways. Table 3 shows the equations and kinetic parameters of pseudo-first-order, pseudo-second-order, and intra-particle diffusion models. The fit quality of the kinetic plots was compared using correlation coefficient.

The pseudo-first-order model suggests that the biosorption process is governed by mass transfer processes, which is caused by fluctuations in the adsorbate concentration between the adsorbent surface and the aqueous solution. The data in Table 3 reveal that this model was not appropriate for the biosorption of copper ions by *A. xylosoxidans*, as supported by low correlation coefficient (*r*^2^ = 0.835; Fig. S4A) and the fact that the experimental value of the biosorption capacity did not coincide with the theoretical value. However, the second-order kinetic model provided a better fit. Furthermore, the pseudo-second-order plot exhibited high linearity with a correlation coefficient value close to 1 (*r*^2^ = 0.999), and the theoretical *q*-value was consistent with the experimental value (Table 3, Fig. S4B). This model proposes that there is a rate-limiting mechanism through the formation of chemical bonds between the metal ions and the adsorbent (Dusengemungu et al., 2020). These results are consistent with earlier investigations on the removal of Cu^2+^ ion by thermally and lyophilized treated bacterial biomass of *Pseudomonas aeruginosa* PAO1 (Kőnig-Peter, Kilar & Pernyeszi, 2019).

The biosorption mechanism was also examined by fitting the kinetic data to the Elovich model (Fig. S4C). The low correlation coefficient (*r*^2^ = 0.811) suggested that this model does not adequately represent the experimental data for Cu^2+^ ion biosorption by bacterial biomass.

Intra-particle diffusion is another significant kinetic model for evaluating the biosorption mechanism. Fig. S4D indicates that the plot of the intra-particle diffusion model is bilinear. The first straight line increases abruptly in a short period of time (*k*_*i*1_ = 5.386 mg/g min^1/2^), indicating that the metal ions quickly penetrate the macropores of the bacterial particles. Subsequently, as shown by the second straight line, the metal ions permeate the mesopores of the bacterial particles more slowly, which is indicated by the lower *k*_*i*2_ value (0.131 mg/g min^1/2^; Table 3). According to Sun et al. (2023), the lower *k*_*i*2_ value compared to *k*_*i*1_ suggests that the external biosorption rate is higher than the intra-particle diffusion rate.

However, the fact that the representation of the intra-particle diffusion model does not cross the origin is important because it means that the overall biosorption kinetics is not controlled by intraparticle diffusion alone. This representation suggests that the biosorption process is a two-zone process, where the biosorption mechanism of copper on *A. xylosoxidans* biomass is influenced by both external film diffusion (zone I) and intra-particle diffusion (zone II). Furthermore, the non-zero values of *C*_*i*1_ (8.675 mg/g) and *C*_*i*2_ (32.51 mg/g) suggest that external diffusion (film diffusion) and interior pore diffusion of metal ions take place simultaneously. Similar findings for copper biosorption by different adsorbents have been reported by other studies (Tran et al., 2017; Kumar & Chawla, 2014).

Based on these data, it can be proposed that the chemical biosorption process governs the biosorption of Cu^2+^ ions by *A. xylosoxidans*, as illustrated by the pseudo-second-order model. Moreover, the biosorption process is determined by a combination of film diffusion and intra-particle diffusion, where film diffusion is the primary factor during the initial phase, while intra-particle diffusion becomes more significant in the subsequent phase, rather than a single rate-limiting step.

### Reuse of the biosorbent

The regeneration efficiency of the biosorbent following metal desorption is crucial to the biotechnological application of biosorption technology for the removal of heavy metals in order to reduce the cost of the process. As shown in Fig. 4, four successive biosorption/desorption experiments were conducted with diluted HCl as an eluent to demonstrate the biosorbent’s reusability. HCl effectively in released copper cations biosorbed through electrostatic attraction *via* proton exchange of copper cations (Buhani et al., 2021). The removal efficiency gradually declined with increasing regeneration cycles. This is attributed to the destruction of active binding sites during the desorption process and losses occurring during repeated washing.

**Figure 4:**
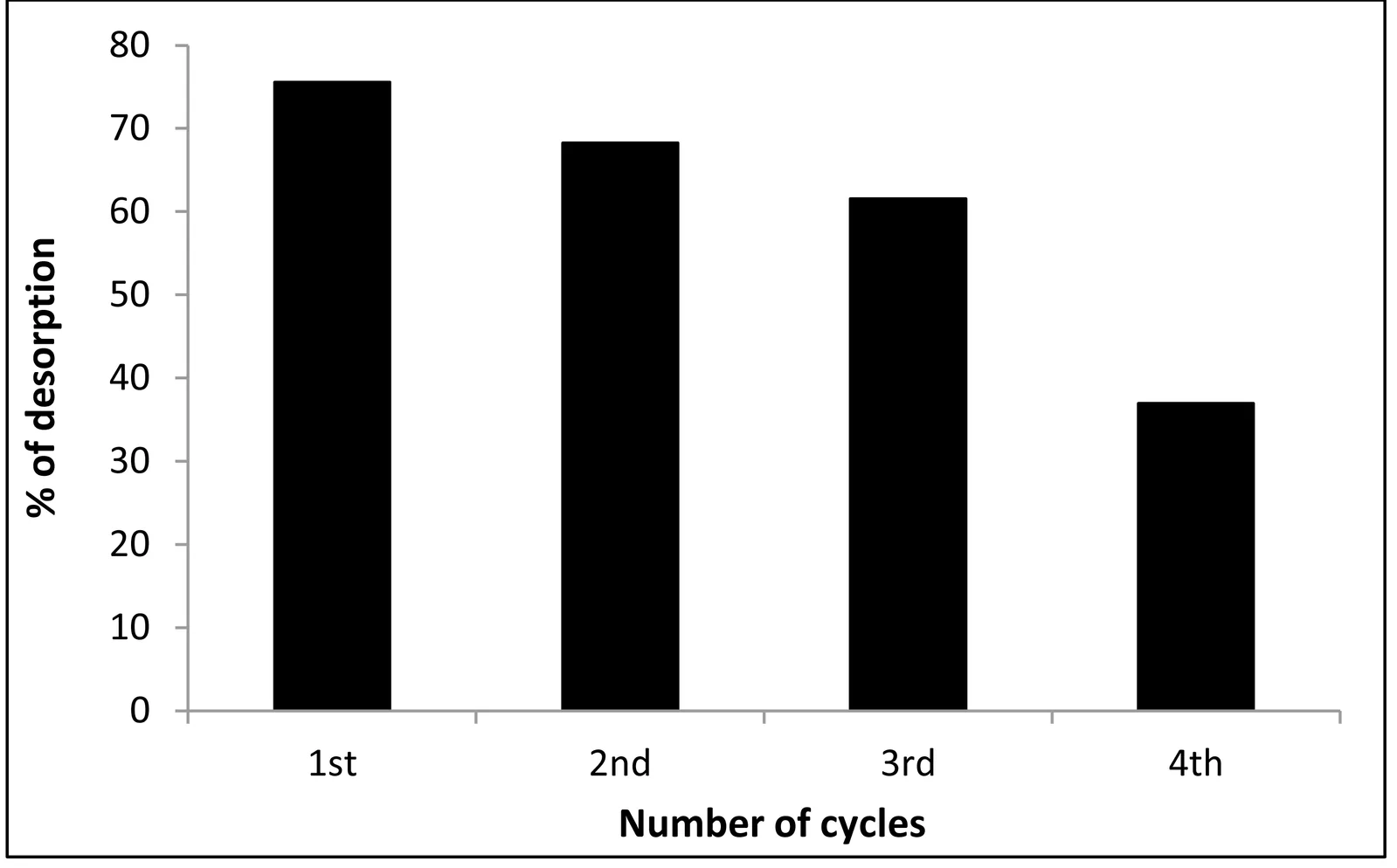
Desorption efficiency of Cu^2+^ from *A. xylosoxidans* using 0.1 M HCl.

The first, second, and third cycles achieved satisfactory Cu^2+^ recovery, with removal efficiencies of 75.55%, 68.26%, and 61.52%, respectively. The findings demonstrated that *A. xylosoxidans* could be recycled for Cu^2+^ up to three times without suffering appreciable sorption performance reductions. Following the third cycle, there was no notable desorption observed, suggesting that a significant fraction of copper ions was irreversibly biosorbed on the bacterial surface. This suggests that chemisorption may substantially contribute to the removal of copper ions (Kulkarni & Kaware, 2015). These results suggest that the *A. xylosoxidans* strain offers a commercial benefit due to its potential for frequent recycling in the biosorption process of copper ions.

### Wastewater treatment applications

Evaluating heavy metal removal from real wastewater is important because it offers a practical evaluation of biosorbent performance compared with standard synthetic solutions. In this study, the wastewater sample indicated temperature, pH, total dissolved solids, and electrical conductivity values of 25.2 °C, 7.6, 23.62 mg/L, and 36.9 µS/cm, respectively. Under optimum conditions, *A. xylosoxidans* achieved a high Cu^2+^ removal efficiency of 88.55%, indicating its efficiency in treating industrial wastewater containing Cu^2+^ ions. Generally, the results contribute to environmental biotechnology by providing a sustainable and scalable technology for the removal of heavy metals, and efficiently bridging the gap between laboratory studies and practical applications by validation using synthetic and real wastewater samples, highlighting the scientific contribution and practical applicability of *A. xylosoxidans* in the treatment of wastewater.

### Biosorption mechanism

The possible biosorption mechanism of *A. xylosoxidans* was determined by comparing the FT-IR spectra of the samples before and after copper biosorption. Figures S5A, S5B displays how copper ions interact with amide and hydroxyl groups by showing a minor shift in the bands of N−H bending vibration of amide group and –OH stretching vibration of polysaccharides from 3,278 cm^−1^ to 3,276 cm^−1^ (Orkusz et al., 2023). According to Dharmapriya et al. (2021), the –CH bending vibration is associated with the biosorption band at 2,927 cm^−1^. The peak at 1730 cm^−1^ disappears during biosorption, indicating that copper binds to carboxyl or carbonyl groups (Waqar et al., 2023). The bending vibration of amide I and amide II of proteins have been attributed to the peaks shifting from 1,633 cm^−1^ to 1,613 cm^−1^ and from 1,539 cm^−1^ to 1,513 cm^−1^, respectively, after copper biosorption on the surface of bacterial biomass (Mecozzi et al., 2012). Additionally, the shift of the peak from 1,451 cm^−1^ to 1,414 cm^−1^ after Cu^2+^biosorption is attributed to the bending vibration of the anhydride –C = O group.

There are multiple overlapping peaks in the range of 1,200 to 1,500 cm^−1^. According to Mecozzi et al. (2012), these bands belong to the stretching frequencies of the –CO groups of free and esterified fatty acids (around 1,259 cm^−1^) and the –CH and –CH_2_ groups of alkyl structures (around 1,382 cm^−1^). Finally, the bands of–C–O–C groups (at 1,131 cm^−1^) and the –CO groups (at 1,052 cm^−1^) of carbohydrates and polysaccharides are located in the range of 1,000 cm^−1^ and 1,250 cm^−1^.

According to these findings, the biosorption of copper ions shifts some wavelengths or causes them to disappear after biosorption. The shift in the intensity of some bands in the FT-IR spectra may be used to suggest possible biosorption mechanism of copper ions. For example, potential hydrogen bonding between Cu^2+^ ions and bacterial biomass was demonstrated by the shift of −OH bending vibration. Furthermore, the interaction of copper ions with N–H groups or some functional groups containing oxygen such as anhydride, carboxyl and carbonyl groups also resulted in an alteration in wavelength, suggesting that complexation is one of the possible mechanisms of the biosorption process (Wang et al., 2015).

The biosorption of Cu^2+^ ions on *A. xylosoxidans* biomass can also be suggested by an ion exchange mechanism, which involves electrostatic interactions of positively charged Cu^2+^ ions with negatively charged functional groups such as hydroxyl (–OH), carbonyl (C=O), and carboxyl (–COOH) (Liu et al., 2023; Banerjee, Bar & Das, 2021). These findings exhibit the close relationship between the surface functional groups of the bacterial biomass and copper ions. Therefore, the biosorption capacity of *A. xylosoxidans* can be enhanced by enriching these functional groups.

To further understand how copper ions are biosorbed on *A. xylosoxidans*, the bacterial biomass was examined using SEM and EDX (Fig. 5).

**Figure 5:**
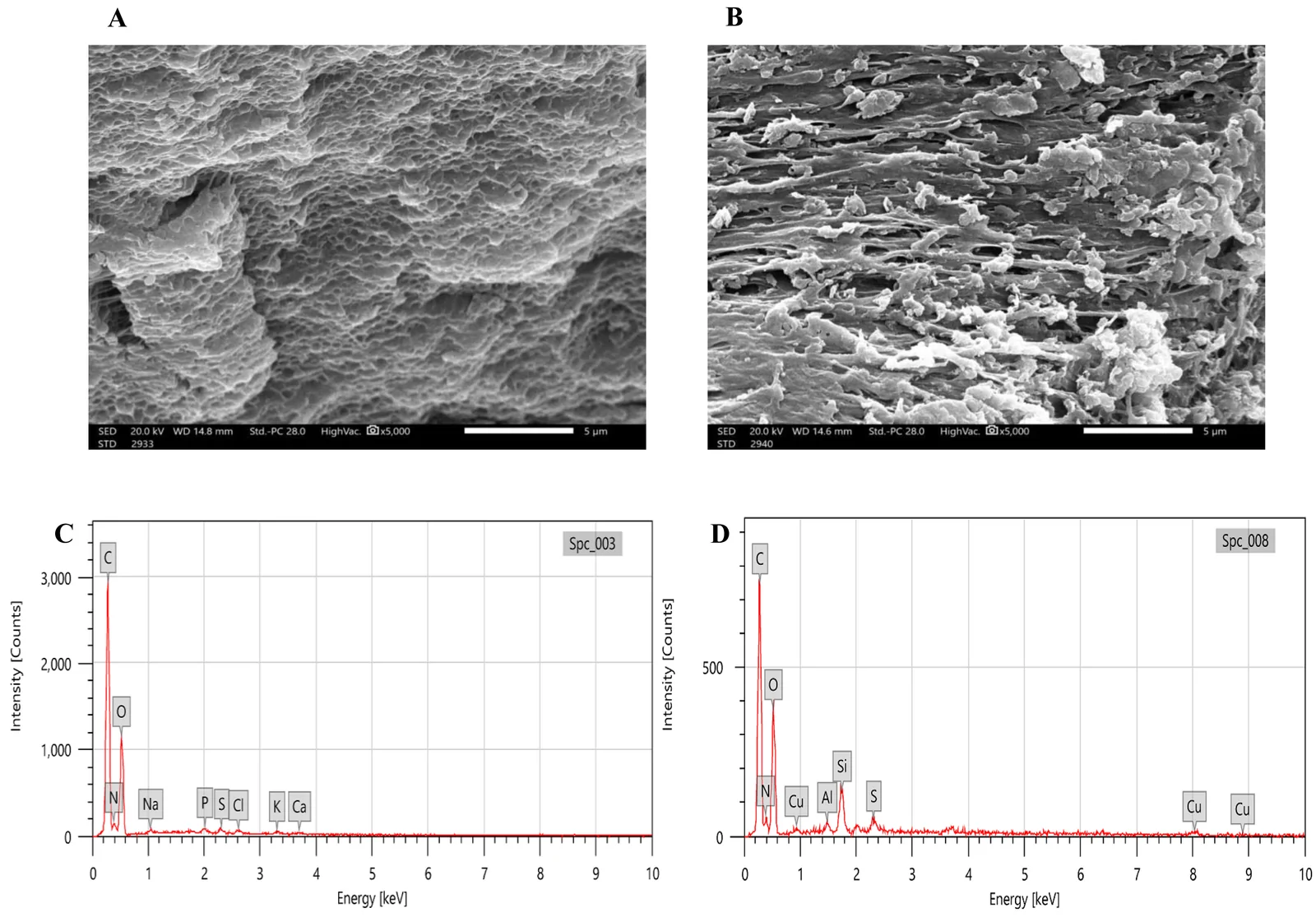
The surface morphology of *A. xylosoxidans* by SEM and EDX analysis before (A, C), and after (B, D) copper ion bioremoval.

Scanning electron microscopy (SEM) images indicated that the biomass surface exhibited a homogeneous structure and pores before copper biosorption (Fig. 5A). These pores may create biosorption channels for copper ions and may improve ion transfer by adhering to the surface of bacteria and dispersing inside the pores. This may be explained by the reduction in surface porosity and the formation of a flat structure after contact with copper ions, as shown in Fig. 5B (Mirghaffari, Moeini & Farhadian, 2015). When the *A. xylosoxidans* bacterial surface was exposed to copper ions, the ions may occupy the vacant biosorption sites and may replace cations on the bacterial surface through a possible ion exchange mechanism (Mata et al., 2008). In addition, some morphological changes caused by the binding of copper ions to functional groups on the surface of *A. xylosoxidans* biomass were observed.

The chemical composition of *A. xylosoxidans* biomass before and after copper biosorption (Figs. 5C, 5D) was evaluated by EDX analysis, and the results revealed that the bacterial biomass contained a high quantity of carbon, nitrogen, oxygen, and silicon, as well as small amounts of sodium, calcium, sulfur, phosphorus, aluminum, chloride, and potassium. Changes in peak intensity were observed in both spectra, suggesting that elemental interactions occurred during the biosorption of copper ions on the bacterial surface. Following the biosorption process, new copper peaks appeared in the EDX spectrum of *A. xylosoxidans* (Fig. 5D). These peaks coincided with the disappearance of peaks for most elements, including potassium, calcium, and sodium. This may be attributed to chemical biosorption and ion exchange, with copper ions replacing potassium, calcium, and sodium ions during the biosorption process (Qin et al., 2019). The EDX spectrum showed that Cu^2+^ was uniformly distributed on the bacterial surface, proving the effectiveness of the biosorption process.

Based on the above findings, it can be concluded that the mechanism of copper uptake by *A. xylosoxidans* suggests the involvement of potential electrostatic attraction, ion exchange, and complex formation. A schematic diagram illustrating the potential mechanism of copper biosorption by *A. xylosoxidans* is shown in Fig. 6. The application of the pseudo-second-order and Langmuir models to *A. xylosoxidans* also supported these interpretations and suggested that chemical biosorption is the mechanism of copper uptake by bacterial biomass.

**Figure 6:**
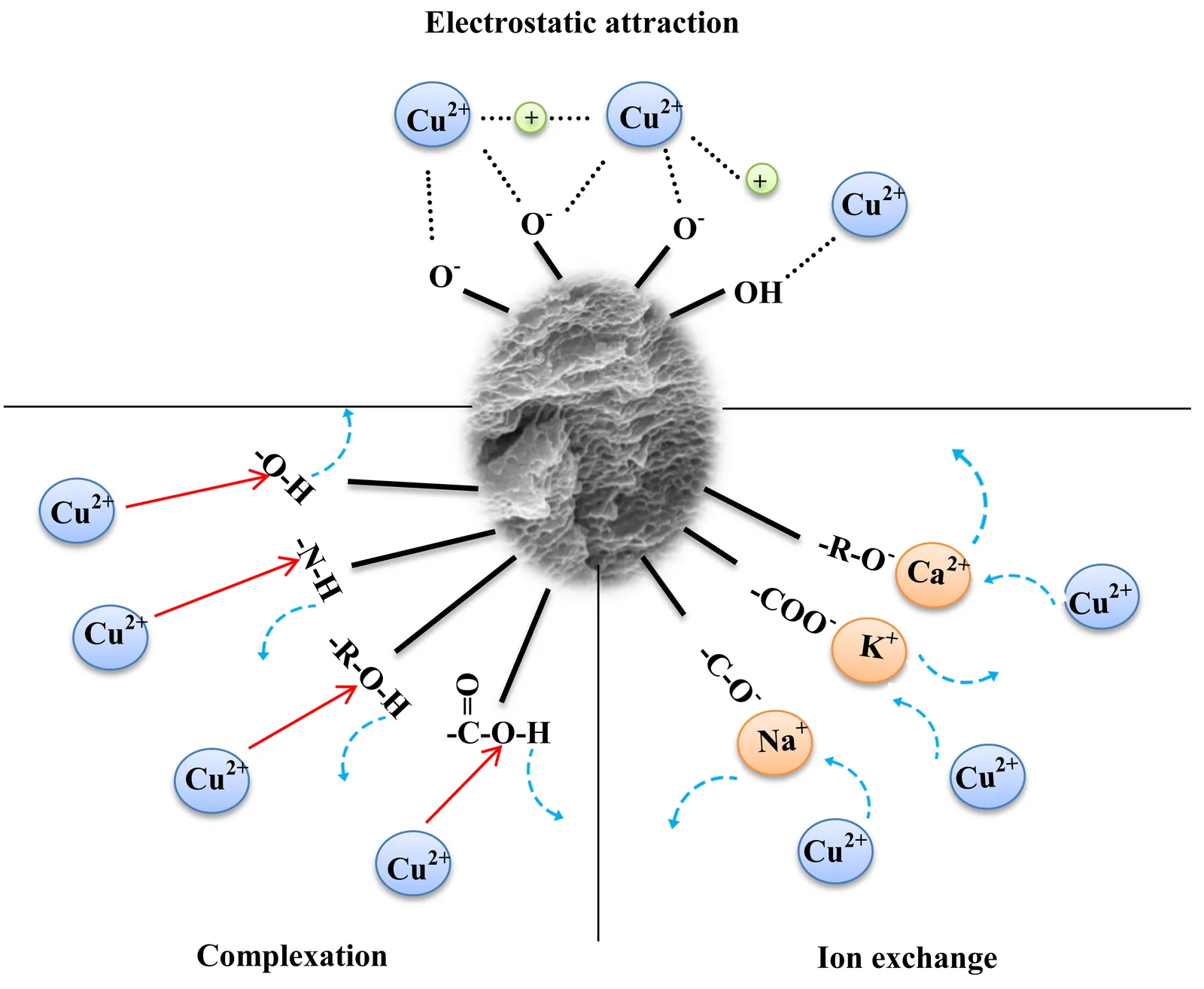
Schematic diagram of the possible mechanism of Cu^2+^ ion biosorption by *A. xylosoxidans*.

### Study strengths, challenges and future prospective

As far as we are aware, no previous study has optimized copper biosorption conditions using RSM/BBD with an indigenous *A. xylosoxidans* strain isolated from waste soil in Jeddah’s Second Industrial City, KSA. The current investigation not only achieves a high biosorption capacity (106.38 mg/g) compared to other bacterial species, but also offers significant innovation by integrating statistical optimization with isotherm, kinetic, thermodynamic, regeneration, and possible mechanistic analyses, in addition to validation using real wastewater samples. Regeneration and recycling experiments further confirmed the operational stability and reusability of the biosorbent, highlighting its practical applicability and scalability compared with traditional biosorption investigations. Despite these promising results, some limitations were encountered due to the lack of characterization techniques such as X-ray diffraction analysis (XRD), X-ray photoelectron spectroscopy (XPS), and Brunauer–Emmett–Teller (BET) analysis due to inadequate facilities. However, this was countered by using Fourier-transform infrared spectroscopy (FTIR) to identify the functional groups in charge of the adsorption process and SEM-EDX, which equally provided the elemental composition. Future studies should focus on large-scale application, cost-effectiveness analysis of improved substitutes, biosorption of different metal ions, and several types of wastewater, and the use of more characterization techniques to further validate biosorption mechanisms.

## Conclusions

The findings of this study indicated that *A. xylosoxidans* can be successfully utilized as a biosorbent for Cu^2+^ removal from aquatic environments. Response surface methodology coupled with Box-Behnken design effectively optimized the biosorption conditions, with the highest copper removal achieved at 34.8 °C, 112.35 mg L^−1^ initial Cu^2+^ concentration, and 1.29 g L^−1^ biosorbent dosage. The maximum biosorption capacity reached 106.38 mg/g, and the experimental data showed strong agreement with the Langmuir isotherm, indicating homogeneous biosorption of copper ions onto the bacterial biomass. Kinetic analysis indicated that the pseudo-second-order model best described the biosorption process, suggesting the involvement of a chemisorption mechanism, while intraparticle diffusion was not the sole rate-controlling step. FTIR and SEM-EDX analyses suggested that ion exchange and electrostatic interactions contributed to copper biosorption. The bacterial strain also showed high Cu^2+^ removal efficiency (88.55%) in real wastewater samples, confirming its practical applicability for industrial wastewater treatment. Additionally, the bacterial strain has shown good potential for regeneration and reuse, highlighting its value as an effective and low-cost bio-adsorbent, particularly since it does not require any modification or chemical application, unlike traditional approaches such as chemical precipitation or activated carbon.

## Supplemental Information

10.7717/peerj.21842/supp-1 Supplemental Information 1 Supplemental Figures and Tables.

10.7717/peerj.21842/supp-2 Supplemental Information 2 Raw Data EDX analysis.

10.7717/peerj.21842/supp-3 Supplemental Information 3 Raw Data SEM analysis.

10.7717/peerj.21842/supp-4 Supplemental Information 4 Raw Data FTIR analysis.

10.7717/peerj.21842/supp-5 Supplemental Information 5 Raw Data RSM-BBD.

10.7717/peerj.21842/supp-6 Supplemental Information 6 Raw Data of biosorption experiments.

## Additional Information and Declarations

### Data Availability

The following information was supplied regarding data availability: The raw measurements are available in the Supplemental Files.
