# Supplementary material for: Copper removal from synthetic wastewater using the bacterium Achromobacter xylosoxidans: biosorption equilibrium, kinetic, thermodynamic and optimizing parameters by Box-Behnken design approach
Source: PeerJ. 2026 Oct 28;14:e21842. doi: 10.7717/peerj.21842 (PMC13723197; doi:10.7717/peerj.21842)
Supplement: Supplementary file 1 [file peerj-14-21842-s001.pdf]

Supplementary data

Copper removal from synthetic wastewater using the bacterium *Achromobacter xylosoxidans*: Biosorption equilibrium, kinetic, thermodynamic and optimizing parameters by Box-Behnken design approach

Mustafa A. Fawzy[1, 2], Mona Alharbi[3], Hana Sonbol[3], Sedky H. A. Hassan[4]*, Mostafa Koutb[5], Mohammed Alorabi[6], Amany G. Ibrahim[1,7]*

[1] Botany and Microbiology Department, Faculty of Science, Assiut University, Assiut 71516, Egypt

[2] Biology Department, College of Sciences, Taif University, P.O. Box 11099, Taif 21944, Saudi Arabia

[3] Department of Biology, College of Science, Princess Nourah bint Abdulrahman University, P.O. Box 84428, Riyadh 11671, Saudi Arabia

[4] Department of Biology, College of Science, Sultan Qaboos University, Muscat 123, Oman

[5] Department of Biology, Faculty of Science, Umm Al-Qura University, Makkah 24381, Saudi Arabia

[6] Biotechnology Department, College of Sciences, Taif University, P.O. Box 11099, Taif 21944, Saudi Arabia

[7] Botany Department, Faculty of Women for Arts, Science and Education, Ain Shams University, Cairo 11566, Egypt

***** Correspondence: s.hassan@squ.edu.om; Tel: +96893879120; amany.g@tu.edu.sa; Tel: +966561479806

Table S1. Box–Behnken Design matrix for the response of copper ions bioremoval by *A. xylosoxidans*

Run order	Actual and coded values			R (%)
	A- Cu^{2+} conc. (mg/L)	B- Bacterial dose (g/L)	C- Temperature (°C)	
1	10(−1)	1.25(−1)	30(0)	78.10
2	120(+1)	1.25(−1)	30(0)	82.40
3	10(−1)	5(+1)	30(0)	71.80
4	120(+1)	5(+1)	30(0)	79.08
5	10(−1)	3.13(0)	20(−1)	69.40
6	120(+1)	3.13(0)	20(−1)	68.83
7	10(−1)	3.13(0)	40(+1)	64.40
8	120(+1)	3.13(0)	40(+1)	74.00
9	65(0)	1.25(−1)	20(−1)	71.08
10	65(0)	5(+1)	20(−1)	74.66
11	65(0)	1.25(−1)	40(+1)	79.06
12	65(0)	5(+1)	40(+1)	71.23
13	65(0)	3.13(0)	30(0)	70.63
14	65(0)	3.13(0)	30(0)	66.08
15	65(0)	3.13(0)	30(0)	65.87

Table S2. Results of ANOVA for copper ions bioremoval by *A. xylosoxidans*

Source	Sum of squares	Degree of freedom	Mean squares	*F*	*p*	
Model	373.02	9	41.45	10.22	0.009	Significant
Residual	20.28	5	4.06	-		
Lack of Fit	5.80	3	1.93	0.267	0.847	Non-significant
Pure Error	14.48	2	7.24	-	-	
Correlation Total	393.30	14	-	-	-	
R^2= 0.948, Adj. R^2= 0.856, Pred. R^2= 0.681, Adeq. Precision= 9.908, CV (%)= 2.781						

Table S3. Desirability of the optimized factors obtained by Box-Behnken Design

Variables	Optimum value			Response		Desirability
	Cu(II) conc. (mg/L)	Bacterial dose (g/L)	Temp. (°C)	Actual R%	Predicted R%	
Values	112.35	1.29	34.80	80.15	82.13	1.00

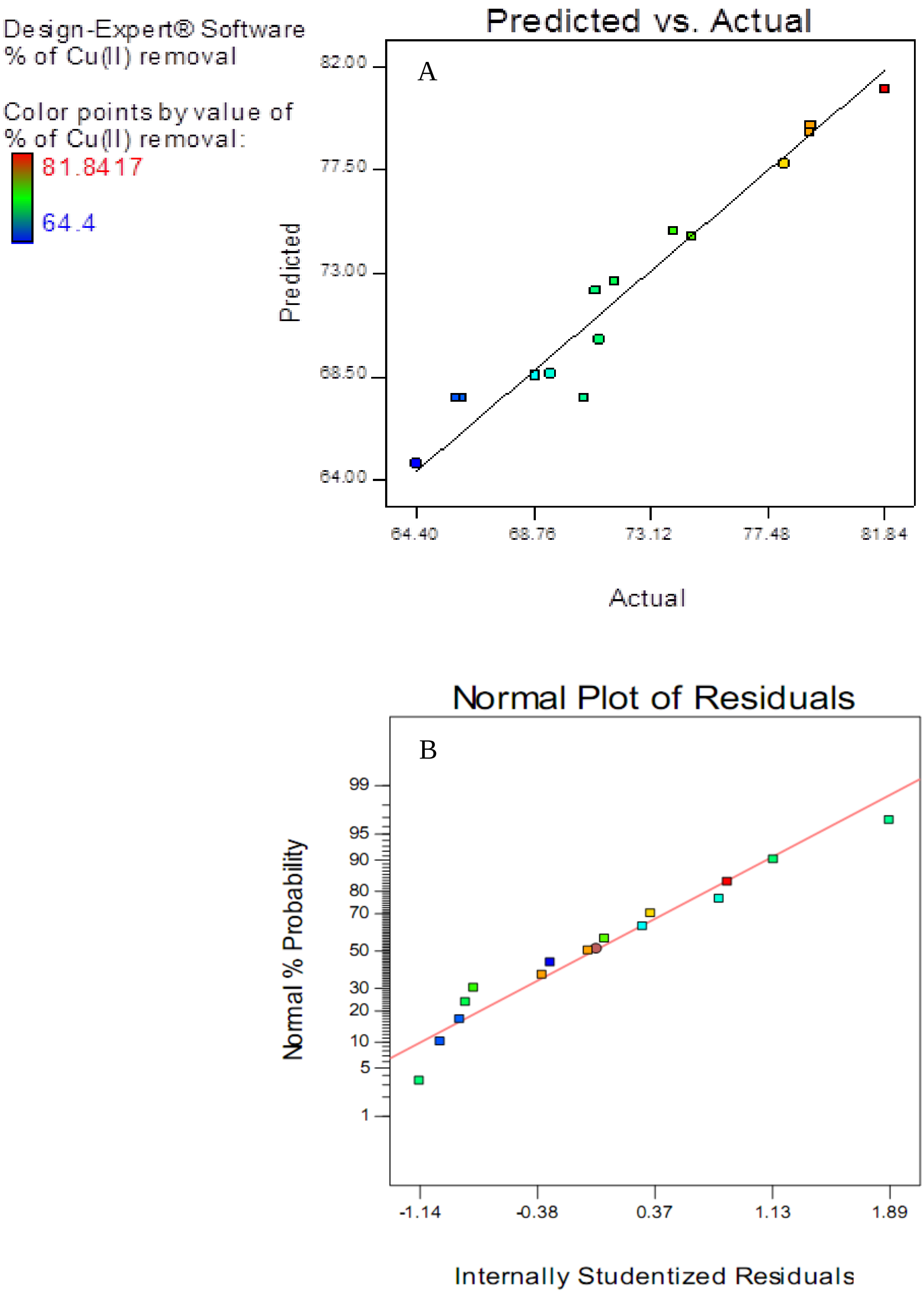

Figure S1. (**A**) The actual versus predicted plot, and **(B)** the normality plot of copper ion bioremoval by *Achromobacter xylosoxidans*

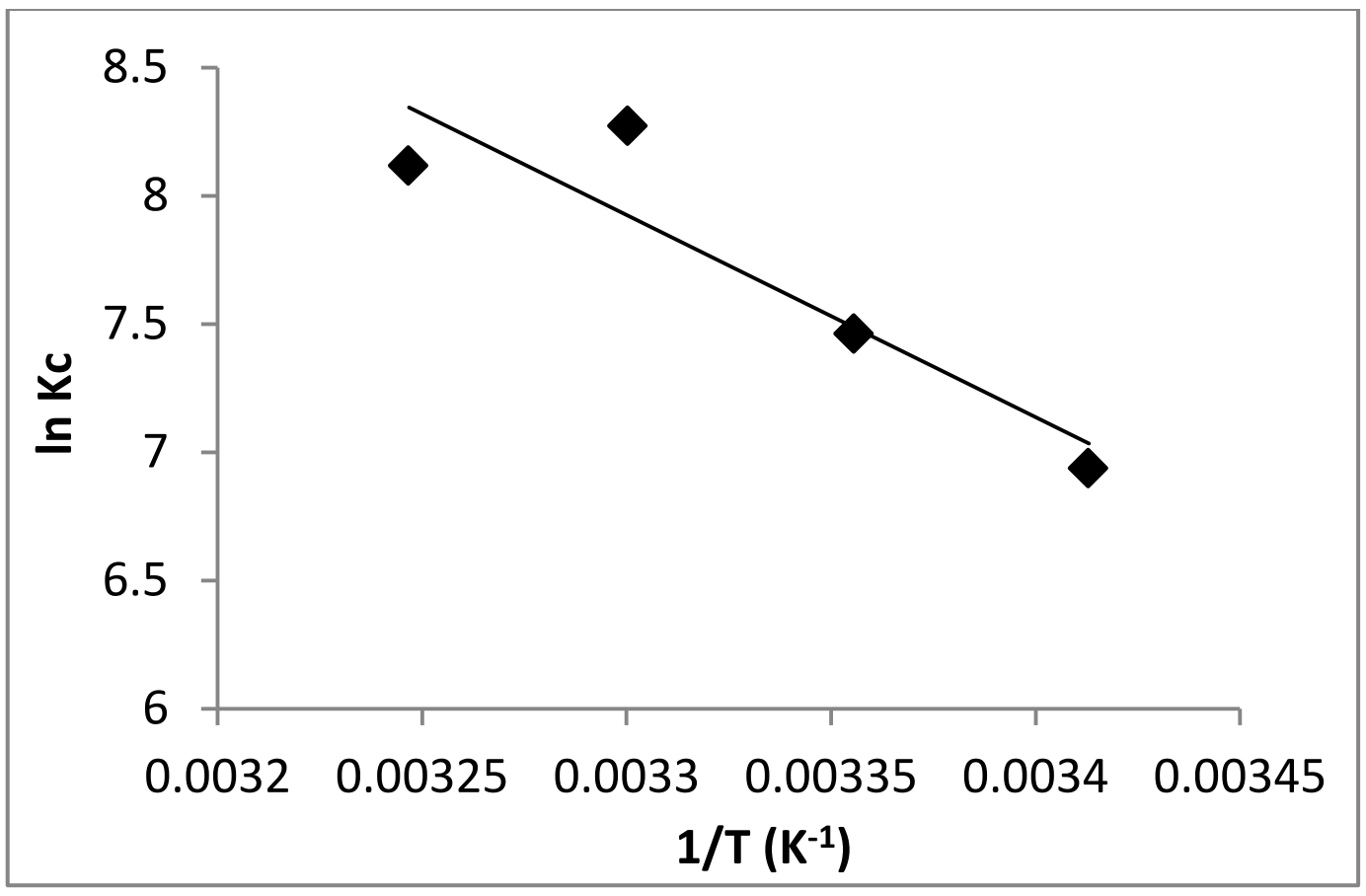

Figure S2. lnKc against 1/T dependency for copper ions bioremoval by *Achromobacter xylosoxidans*.

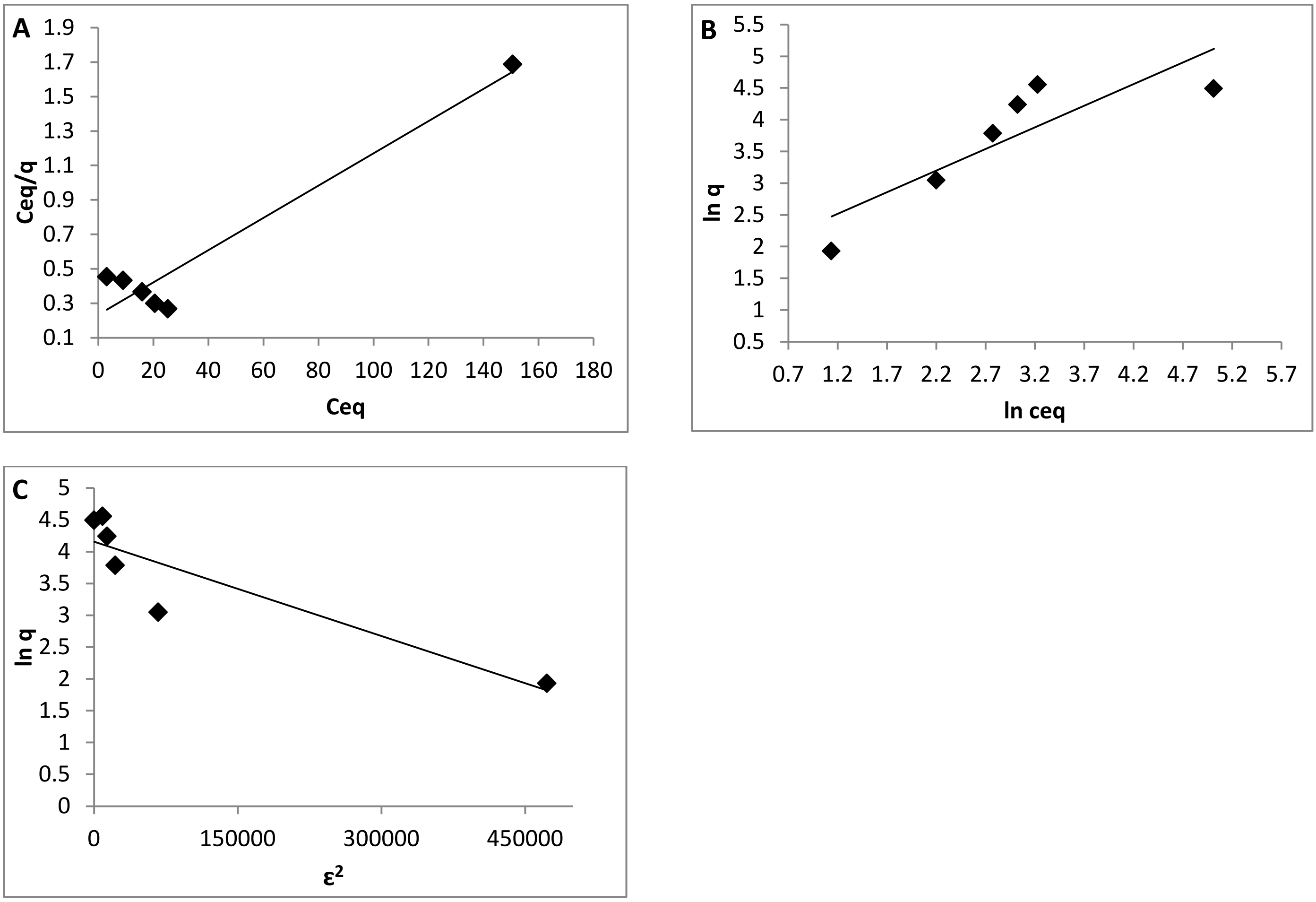

Figure S3. Biosorption isotherms of copper ions by *A. xylosoxidans*: **(A)** Langmuir, **(B)** Freundlich, and **(C)** Dubinin-Radushkevich models.

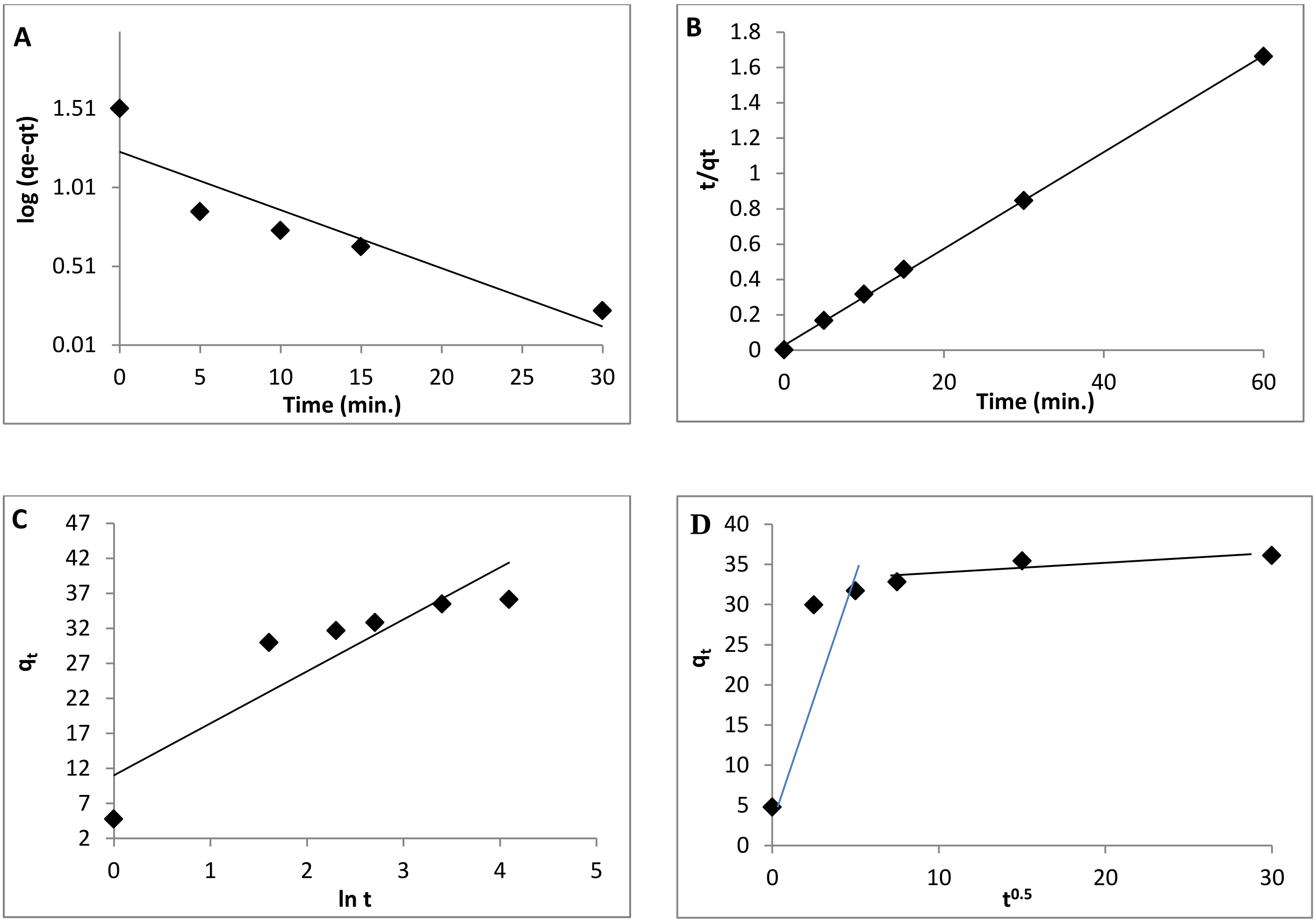

Figure S4. Kinetic models of copper ions by *A. xylosoxidans*: **(A)** pseudo–first–order, **(B)** pseudo–second–order, **(C)** Elovich, and **(D)** Intra-particle diffusion models.

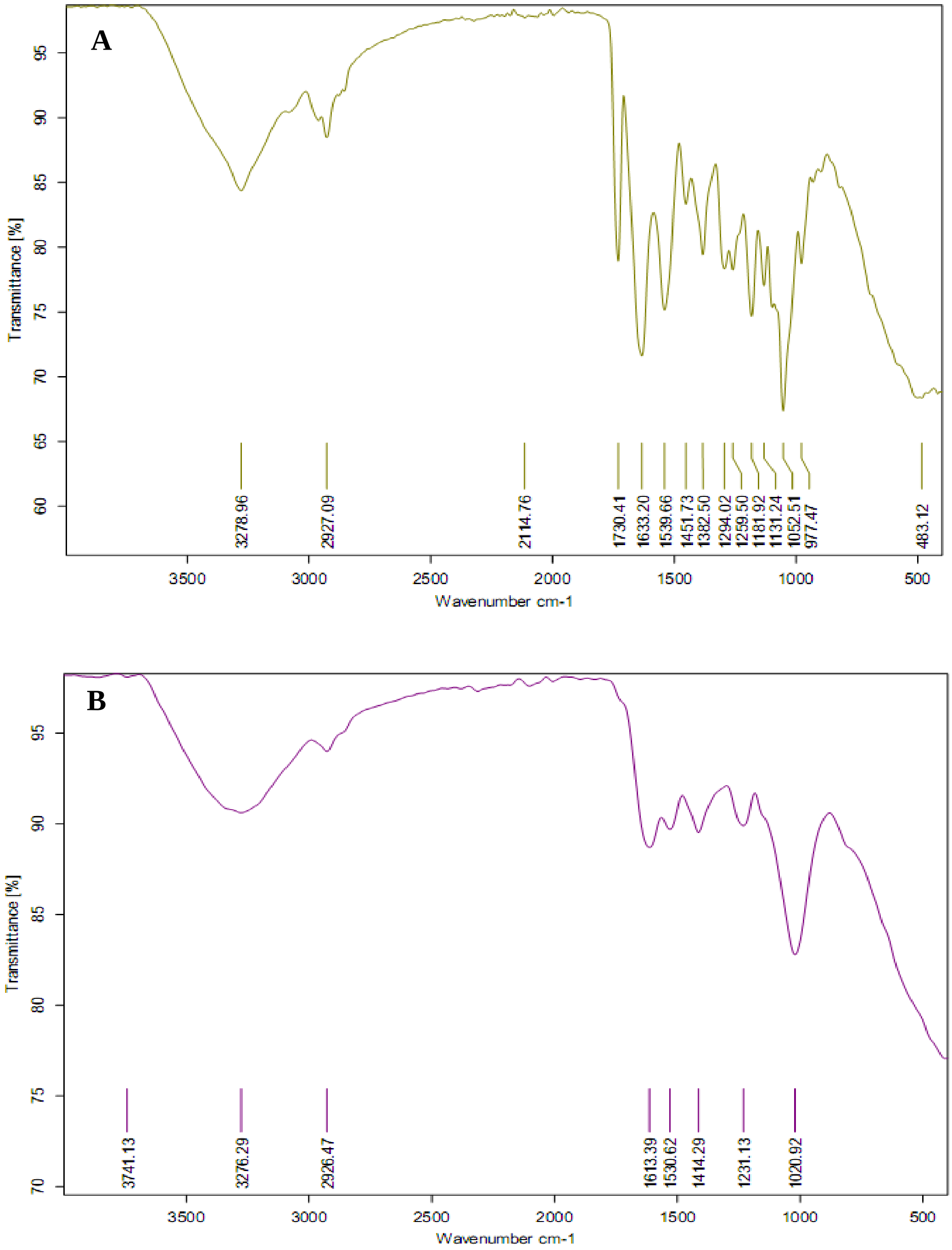

Figure S5. FTIR spectra of *A. xylosoxidans* biomass before **(A)**, and after **(B)** copper ion bioremoval.
