# Supplementary material for: Copper removal from synthetic wastewater using the bacterium Achromobacter xylosoxidans: biosorption equilibrium, kinetic, thermodynamic and optimizing parameters by Box-Behnken design approach
Source: PeerJ. 2026 Oct 28;14:e21842. doi: 10.7717/peerj.21842 (PMC13723197; doi:10.7717/peerj.21842)
Supplement: Supplementary file 2 [file peerj-14-21842-s002.pdf]

Electron Microscope Unit
Faculty Of Science
Alexandria University

Lab's Logo or identification mark

EGAC Accredited testing CAB #220020

Sem_SED_018

Signal SED
Landing Voltage 20.0 kV
WD 10.0 mm
Magnification x600
Vacuum Mode HighVacuum

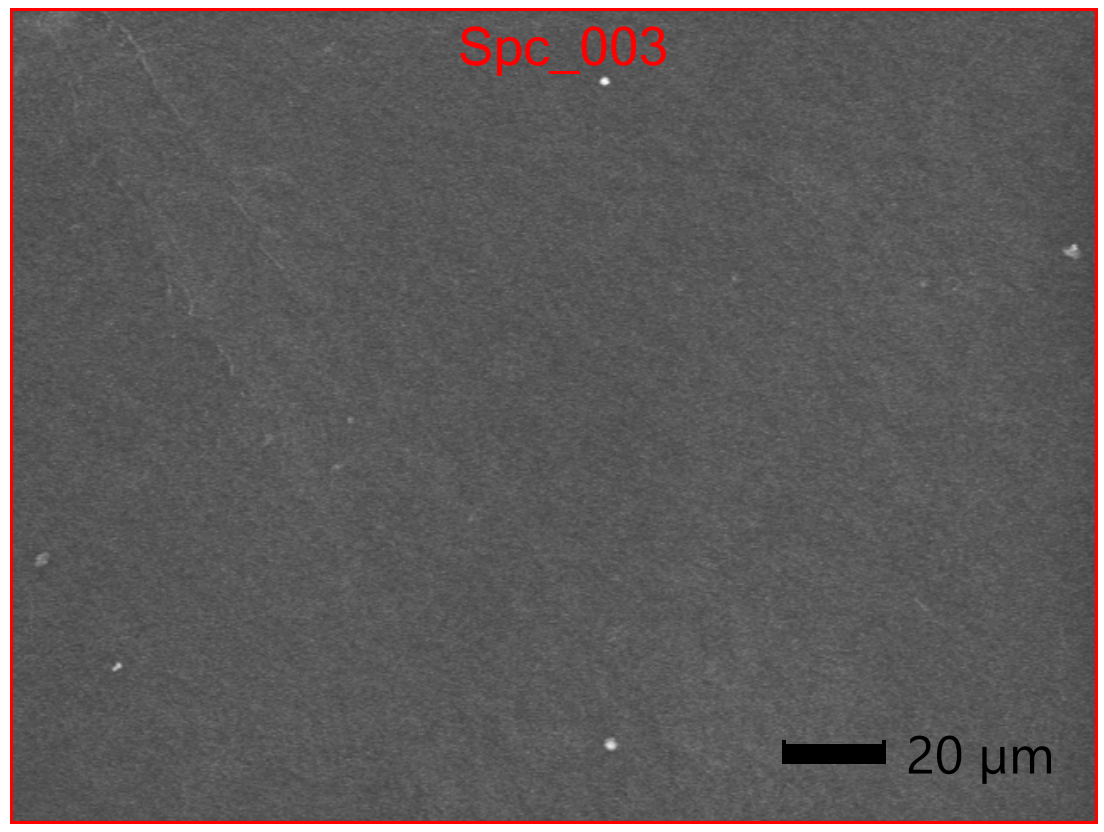

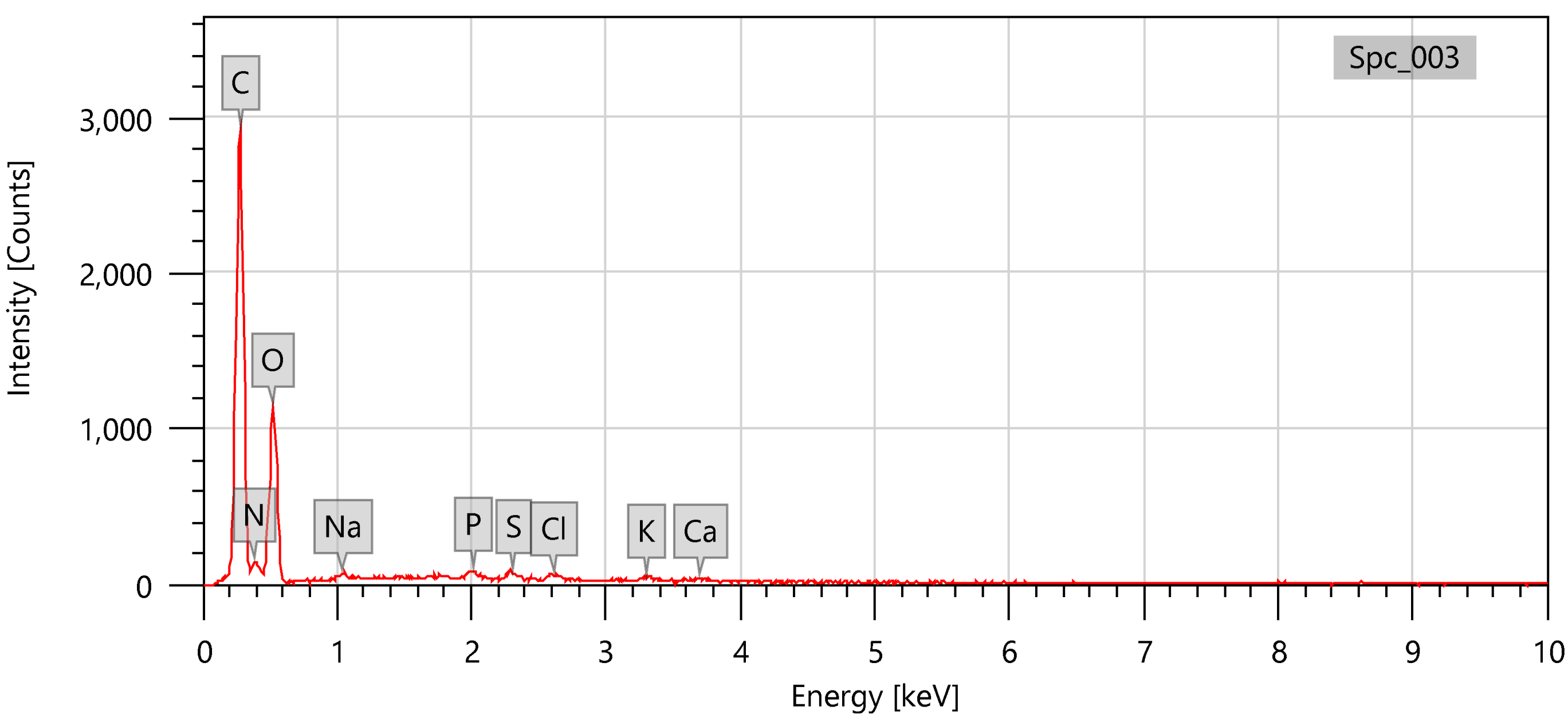

Items	Value
measurement conditions	
Acceleration voltage	20.00 kV
Probe current	-
Magnification	x 600
Process time	T3
Measurement detector	First
Live time	30.00 seconds
Real time	30.48 seconds
Dead time	2.00 %
Count rate	1551.00 CPS

Display name	Standard data	Quantification method	Result Type
Spc_003	Standardless	ZAF	Metal

Element	Line	Mass%	Atom%
C	K	50.57±0.21	57.47±0.23
N	K	8.89±0.35	8.67±0.34
O	K	38.82±0.45	33.12±0.38
Na	K	0.35±0.04	0.21±0.02
P	K	0.35±0.03	0.15±0.01
S	K	0.32±0.02	0.14±0.01
Cl	K	0.29±0.02	0.11±0.01
K	K	0.28±0.03	0.10±0.01
Ca	K	0.13±0.02	0.04±0.01
Total		100.00	100.00
Spc_003			Fitting ratio 0.0252

Lab's Logo or identification mark

EGAC Accredited testing CAB #220020

Electron Microscope Unit
Faculty Of Science
Alexandria University

Sem_SED_023

Signal SED
Landing Voltage 20.0 kV
WD 10.0 mm
Magnification x650
Vacuum Mode HighVacuum

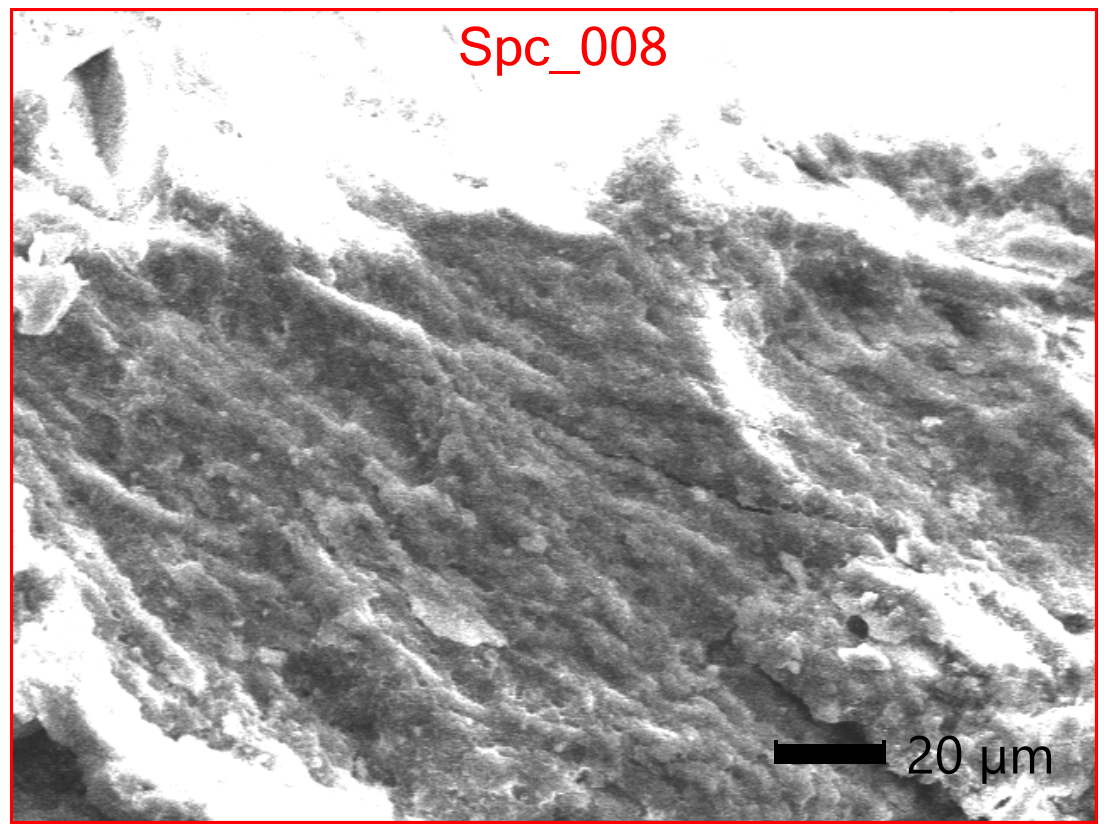

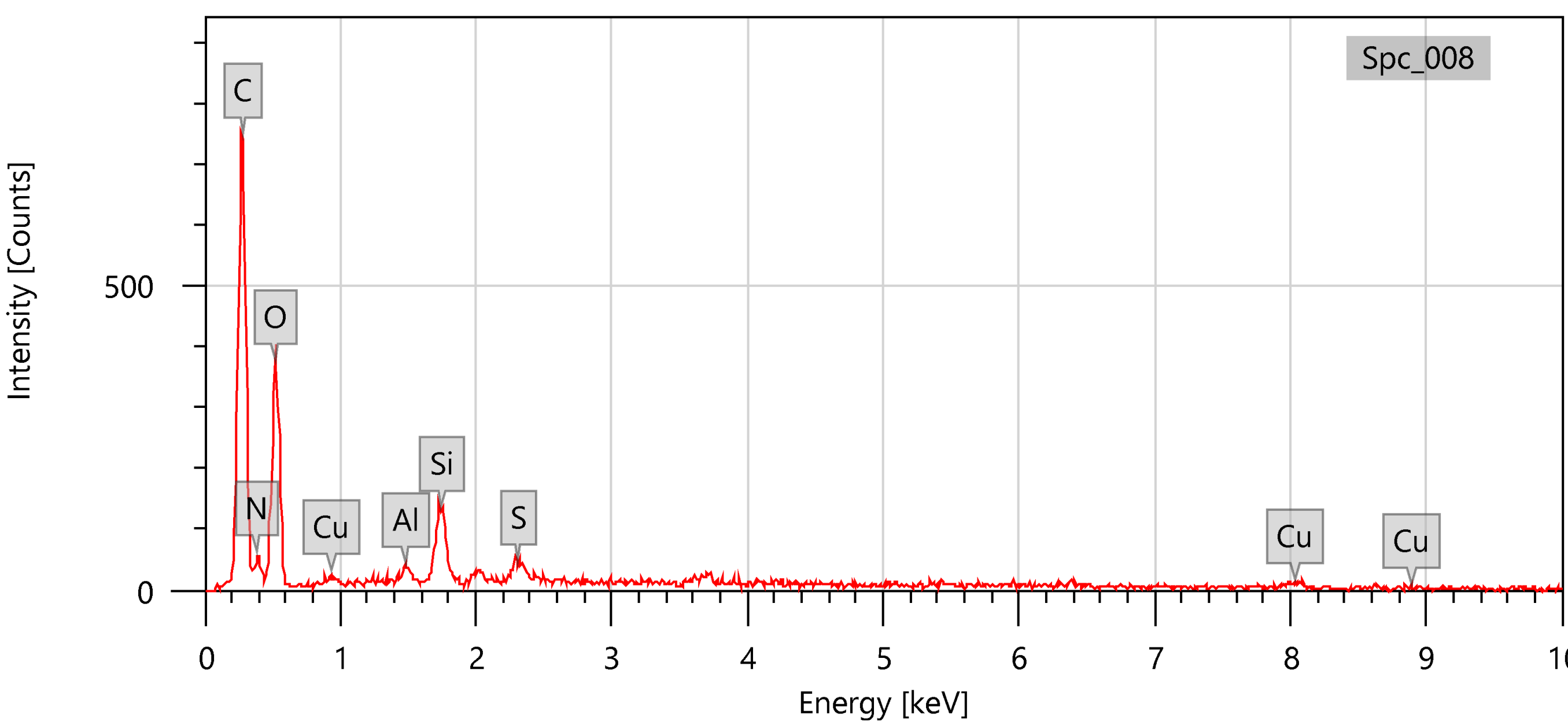

Items	Value
measurement conditions	
Acceleration voltage	20.00 kV
Probe current	-
Magnification	x 650
Process time	T3
Measurement detector	First
Live time	30.00 seconds
Real time	30.49 seconds
Dead time	2.00 %
Count rate	635.00 CPS

Display name	Standard data	Quantification method	Result Type
Spc_008	Standardless	ZAF	Metal

Element	Line	Mass%	Atom%
C	K	46.57±0.37	54.22±0.43
N	K	11.21±0.61	11.19±0.61
O	K	37.35±0.76	32.65±0.66
Al	K	0.47±0.05	0.24±0.03
Si	K	2.10±0.10	1.04±0.05
S	K	0.68±0.06	0.30±0.03
Cu	K	1.62±0.18	0.36±0.04
Total		100.00	100.00
Spc_008			Fitting ratio 0.0803
