# Supplementary material for: Copper removal from synthetic wastewater using the bacterium Achromobacter xylosoxidans: biosorption equilibrium, kinetic, thermodynamic and optimizing parameters by Box-Behnken design approach
Source: PeerJ. 2026 Oct 28;14:e21842. doi: 10.7717/peerj.21842 (PMC13723197; doi:10.7717/peerj.21842)
Supplement: Supplementary file 3 [file peerj-14-21842-s003.pdf]

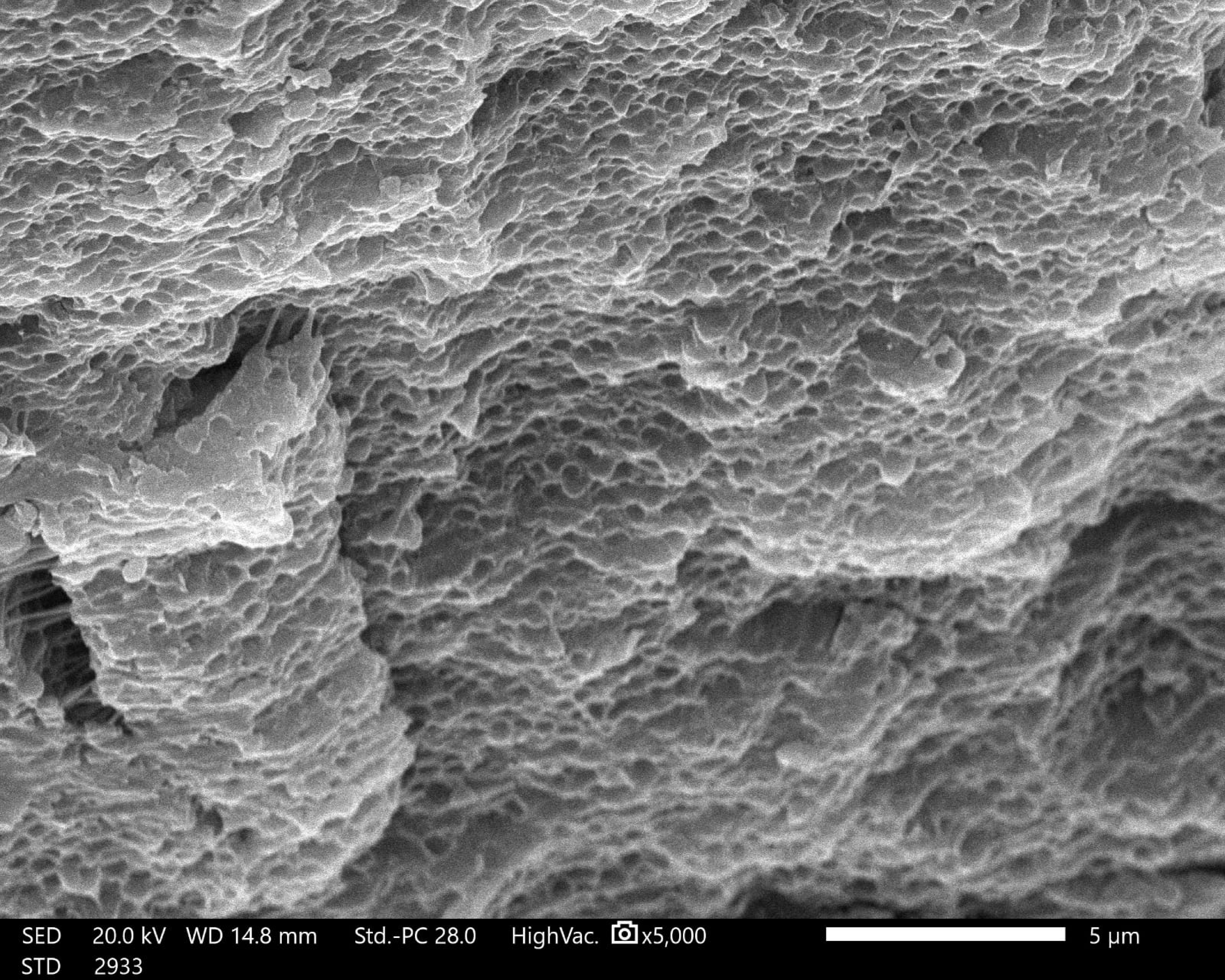
SED 20.0 kV WD 14.8 mm Std.-PC 28.0 HighVac. x5,000
5 μm
STD 2933

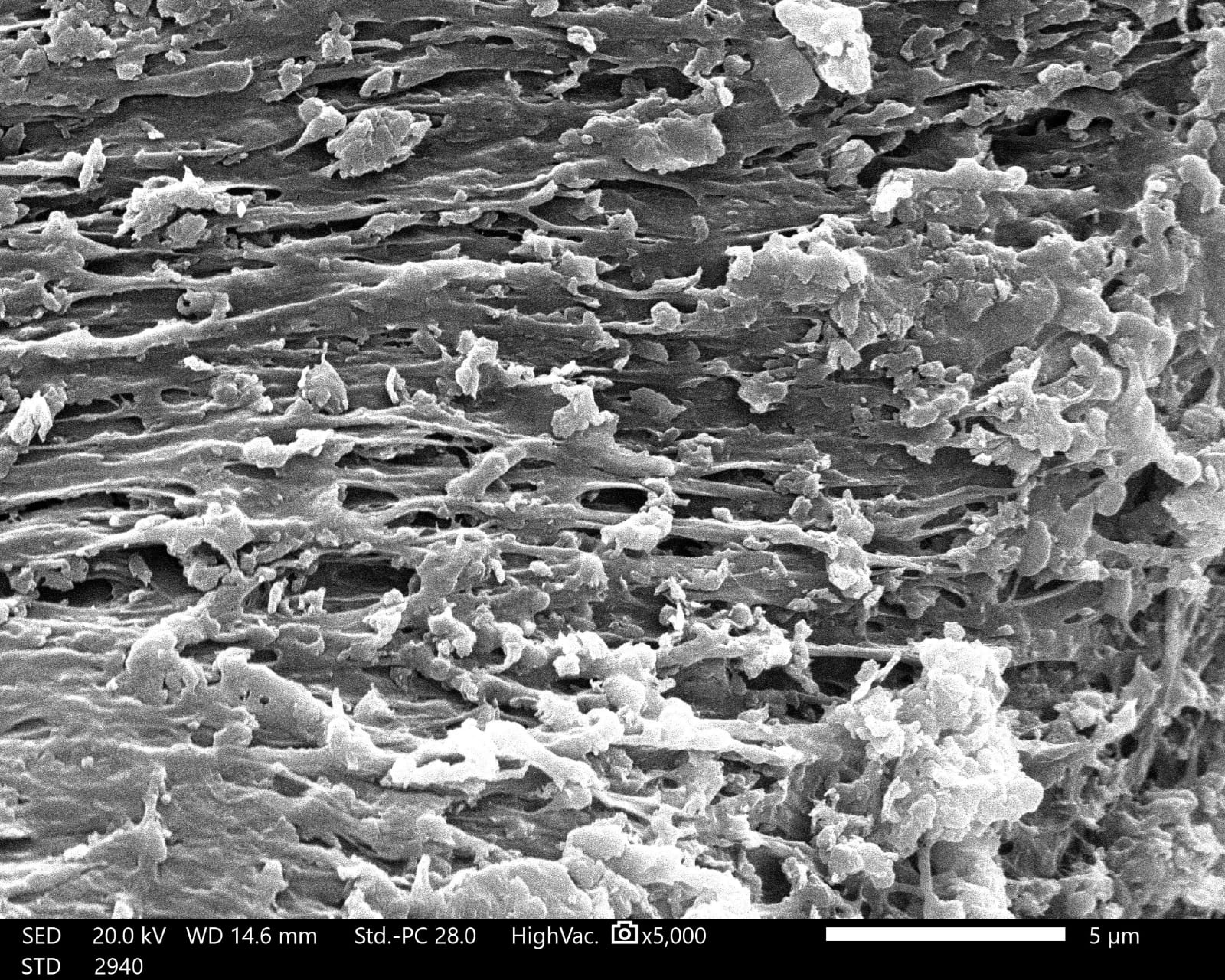
SED
20.0 kV
WD 14.6 mm
Std.-PC 28.0
HighVac.
x5,000
5 µm
STD
2940
