# Supplementary material for: Copper removal from synthetic wastewater using the bacterium Achromobacter xylosoxidans: biosorption equilibrium, kinetic, thermodynamic and optimizing parameters by Box-Behnken design approach
Source: PeerJ. 2026 Oct 28;14:e21842. doi: 10.7717/peerj.21842 (PMC13723197; doi:10.7717/peerj.21842)
Supplement: Supplementary file 4 [file peerj-14-21842-s004.pdf]

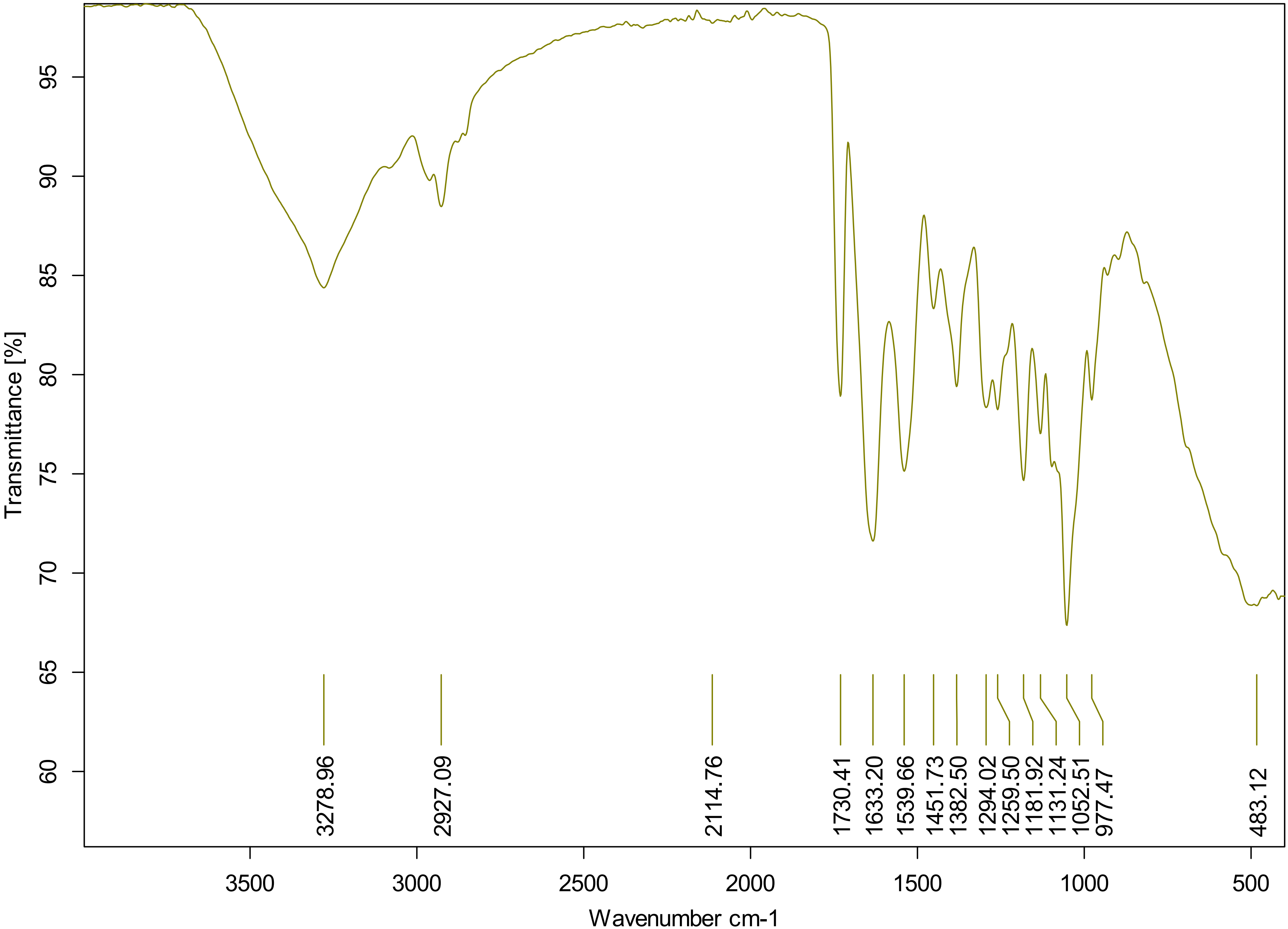
95
90
85
80
75
70
65
60
Transmittance [%]
3278.96
2927.09
2114.76
1730.41
1633.20
1539.66
1451.73
1382.50
1294.02
1259.50
1181.92
1131.24
1052.51
977.47
483.12
3500
3000
2500
2000
1500
1000
500
Wavenumber cm-1

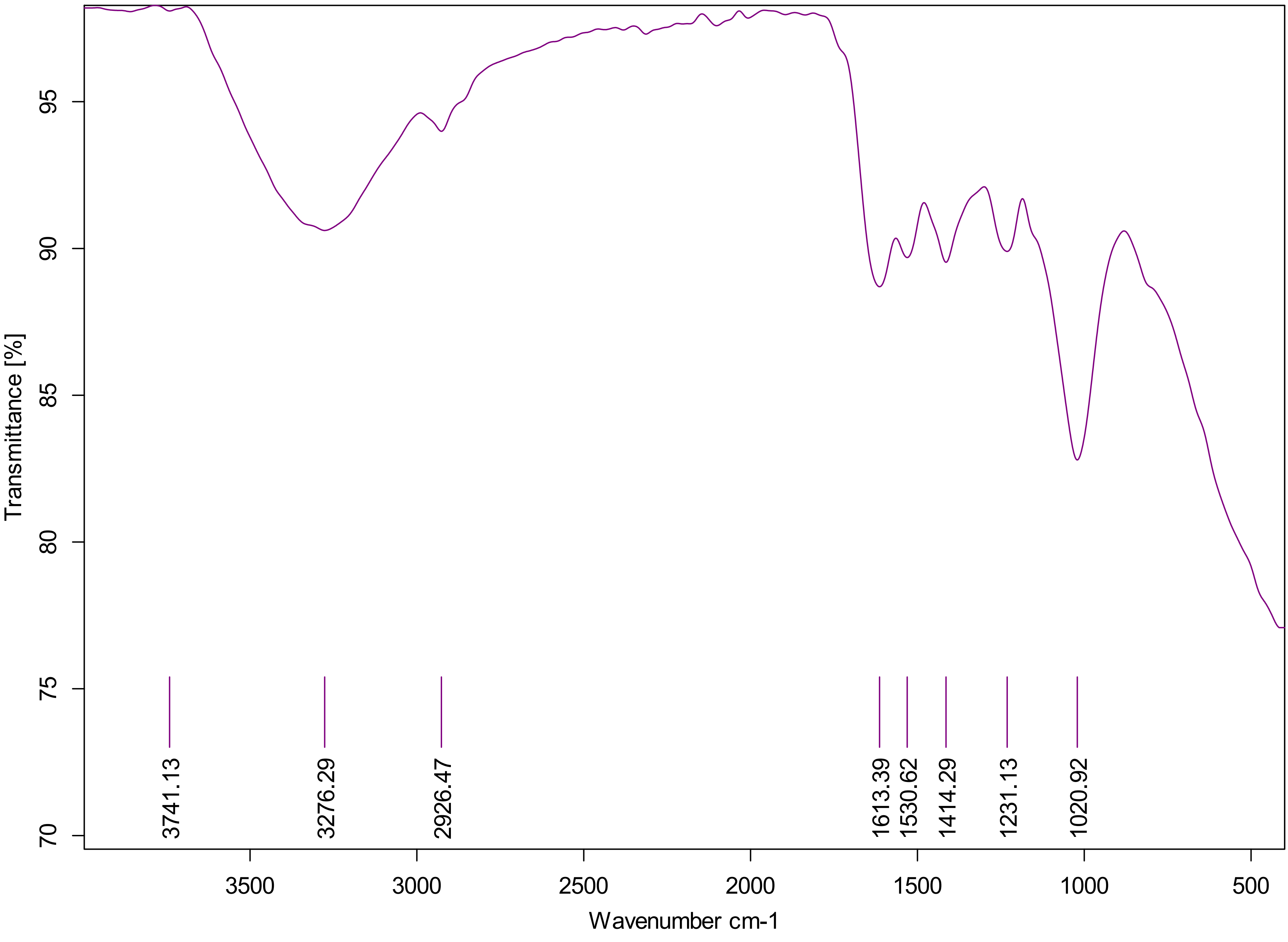
95
90
85
80
75
70
Transmittance [%]
3741.13
3276.29
2926.47
1613.39
1530.62
1414.29
1231.13
1020.92
3500
3000
2500
2000
1500
1000
500
Wavenumber cm-1
